# Dynamic Transformation of Nano-MoS_2_ in a Soil–Plant System Empowers Its Multifunctionality on Soybean Growth

**DOI:** 10.1021/acs.est.3c09004

**Published:** 2024-01-04

**Authors:** Mingshu Li, Peng Zhang, Zhiling Guo, Weichen Zhao, Yuanbo Li, Tianjing Yi, Weidong Cao, Li Gao, Chang Fu Tian, Qing Chen, Fazheng Ren, Yukui Rui, Jason C. White, Iseult Lynch

**Affiliations:** † Department of Environmental Science and Engineering, University of Science and Technology of China, Hefei 230026, China; ‡ College of Resources and Environmental Sciences, China Agricultural University, Beijing 100193, China; § China CDC Key Laboratory of Environment and Population Health, National Institute of Environmental Health, Chinese Center for Disease Control and Prevention, Beijing 100021, China; ∥ School of Geography, Earth and Environmental Sciences, University of Birmingham, Edgbaston, Birmingham B15 2TT, U.K.; ⊥ State Key Laboratory of Environmental Chemistry and Ecotoxicology, Research Center for Eco-Environmental Sciences, Chinese Academy of Sciences, Beijing 100085, China; # Institute of Agricultural Resources and Regional Planning, Chinese Academy of Agricultural Sciences, Beijing 100081, China; ∇ State Key Laboratory for Biology of Plant Disease and Insect Pests, Institute of Plant Protection, Chinese Academy of Agricultural Sciences, Beijing 100193, China; ○ State Key Laboratory of Agrobiotechnology, College of Biological Sciences, China Agricultural University, Beijing 100193, China; ◆ Key Laboratory of Precision Nutrition and Food Quality, China Agricultural University, Beijing 100083, China; ¶ The Connecticut Agricultural Experiment Station, New Haven, Connecticut 06504, United States

**Keywords:** MoS_2_ nanoparticles, soybean, biodistribution, biotransformation

## Abstract

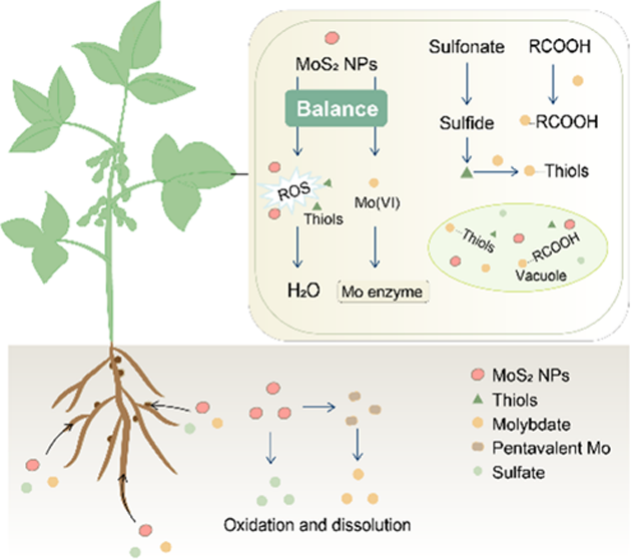

Molybdenum disulfide (nano-MoS_2_) nanomaterials have shown great potential for biomedical and catalytic applications due to their unique enzyme-mimicking properties. However, their potential agricultural applications have been largely unexplored. A key factor prior to the application of nano-MoS_2_ in agriculture is understanding its behavior in a complex soil–plant system, particularly in terms of its transformation. Here, we investigate the distribution and transformation of two types of nano-MoS_2_ (MoS_2_ nanoparticles and MoS_2_ nanosheets) in a soil–soybean system through a combination of synchrotron radiation-based X-ray absorption near-edge spectroscopy (XANES) and single-particle inductively coupled plasma mass spectrometry (SP-ICP-MS). We found that MoS_2_ nanoparticles (NPs) transform dynamically in soil and plant tissues, releasing molybdenum (Mo) and sulfur (S) that can be incorporated gradually into the key enzymes involved in nitrogen metabolism and the antioxidant system, while the rest remain intact and act as nanozymes. Notably, there is 247.9 mg/kg of organic Mo in the nodule, while there is only 49.9 mg/kg of MoS_2_ NPs. This study demonstrates that it is the transformation that leads to the multifunctionality of MoS_2_, which can improve the biological nitrogen fixation (BNF) and growth. Therefore, MoS_2_ NPs enable a 30% increase in yield compared to the traditional molybdenum fertilizer (Na_2_MoO_4_). Excessive transformation of MoS_2_ nanosheets (NS) leads to the overaccumulation of Mo and sulfate in the plant, which damages the nodule function and yield. The study highlights the importance of understanding the transformation of nanomaterials for agricultural applications in future studies.

## Abstract 2

The transformation of Nano-MoS_2_ in the soil–plant system endows it with multifunctionality to improve soybean growth and yield.

## Introduction

Mo is an essential micronutrient for plant growth and development, especially for legume plants, which need a large number of nitrogen nutrients for their growth. This is due to the fact that Mo is the metal center of the nitrogenase, which is an essential enzyme responsible for BNF, a process by which legume plants transform N_2_ from the air into ammonium for plant use through symbiotic relationships with nitrogen-fixing bacteria in their nodules.^1^ In addition to nitrogenase, Mo is also involved in several essential enzymes that participate in nitrogen metabolism, such as nitrate reductase, which converts nitrate (NO_3_^–^) to nitrite (NO_2_^–^) during the process of nitrate assimilation.^2^ Mo is a cofactor for this enzyme and is required for its activity. Mo is also the cofactor of xanthine dehydrogenase, which catalyzes xanthine and hypoxanthine to produce uric acid. The other two enzymes involving Mo are sulfite oxidase, which catalyzes the oxidation of sulfite to sulfate, and aldehyde oxidase, which catalyzes the oxidation of aldehydes to carboxylic acids.

Although Mo is a micronutrient for plants and Mo deficiency in plants is rare as the amount of Mo in soil is usually sufficient, when it does occur, it can cause severe damage to both plant growth and yield, especially in legume plants.^3^ Supplementing with Mo (e.g., Na_2_MoO_4_ fertilizer) has been shown to significantly stimulate plant growth and yield. However, the efficiency of Mo fertilizer use in plants can vary depending on soil properties, such as pH, organic matter content, and plant species. In alkaline soils, Mo is less available to plants, while in soils with low organic matter content, Mo tends to leach out quickly.^4^

Nanotechnology offers new opportunities for sustainable agriculture.^5^ Specifically, producing fertilizers on a nanoscale has shown the potential to enhance the efficiency of traditional fertilizers through target delivery, slow release, or responsive release mechanisms. However, studies on using nanoscale Mo are still scarce. Nanoscale MoO_3_ has been shown to increase nitrate utilization in rice.^6^ Positive effects, such as enhanced nutrient uptake and root area, were also reported in chickpeas after foliar treatment with biosynthesized Mo nanoparticles (composition not reported).^7^ The release of the essential plant nutrient Mo from nano-MoS_2_ undoubtedly contributed to the observed positive effects.^8^ However, another key proposed mechanism attributes part of the effects to the antioxidant enzyme mimic activity of Mo nanoparticles.^9^ Both Mo oxide and sulfide nanomaterials have nanoenzymatic properties, such as peroxidase, superoxide dismutase, or catalase activity, which endow them the capacity to capture excessive reactive oxygen (ROS) species, thus protecting plants against oxidative damage.^10,11^ However, clear evidence especially whether these nanozymes remain in nanoform in the plant life cycle to maintain their enzyme function is lacking.

We argue that the biological effects of Mo nanomaterials on plants can be attributed to the combined effects of both mechanisms. Nanomaterials are dynamic in the environment and their physicochemical properties may change upon entering the environment.^12^ For example, nano-MoS_2_ may oxidize, dissolve, and release MoO_4_^2–^, which is an essential micronutrient for plants.^13,14^ This process is called “transformation”. However, the transformation will “break down” the nanozymes, which seems paradoxical for nanozymes because the enzyme mimetic function can be realized only as “intact” particles. Maintaining a balance between the two mechanisms to meet the requirements of plants at different growth stages seems to be the key to maximizing the benefits of Mo materials. To achieve this, revealing the mechanism of the action of Mo nanomaterials on plants is crucial. This necessitates a comprehensive understanding of the dynamic transformation processes occurring within the plant life cycle, a realm in which our knowledge is currently deficient.

Here, we investigated the transformation of nano-MoS_2_ in a soil–soybean system in a life cycle study. We compared three different sizes of nano-MoS_2_, i.e., MoS_2_ NPs, MoS_2_ NS, and MoS_2_ bulk (Supporting Information Figures 1 and 2). We found distinct effects of the three materials, with the MoS_2_ NPs enhancing yield even at low dosages and without showing toxicity even at high dosages. To explore the mechanism underlying this distinction, we comprehensively analyzed the dynamics of the transformation of MoS_2_ in a soil–soybean system at 30, 60, and 90 days (d) using XANES, dissolution test, as well as SP-ICP-MS. These three time points represent three important growth stages of soybean, which have different physiological characteristics and requirements of nitrogen. By comparing different materials and growth stages, we revealed the mechanisms of the multifunctionality of MoS_2_ NPs that caused enhanced BNF and yield.

## Materials and Methods

### Plant Culture and Exposure

Soil for plant culture was collected from an agricultural field in Beijing (40°14′40.91′′ N; 116°19′17.94′′ E), sieved with a 2 mm sieve, and air-dried. Potting soil was purchased from Scotts Miracle-Gro Products Inc. in the USA and mixed with the dried soil at a volume ratio of 1:1. The properties of the mixed soil are provided in Supporting Information Table 1. MoS_2_ NPs (lateral size: 106.8 nm, thickness: 20.1 nm), MoS_2_ NS (lateral size: 115.6 nm, thickness: 4.3 nm), MoS_2_ bulk (lateral size: 2.6 μm, thickness: 121.1 nm), or Na_2_MoO_4_ was directly added to 500 g of soil. To achieve homogeneity, the soil was mixed with a hand mixer for 5 min and then with a drum mixer for 1 h. The hydrodynamic size and ζ potential of MoS_2_ NPs, MoS_2_ NS, and MoS_2_ bulk are provided in Supporting Information Table 2. The final concentrations were 10, 100, and 500 mg/kg. Untreated soil with no Mo was used as a control.

Soybean seeds (Hedou 13), purchased from Shouguang Seeds & Seedling Co., Ltd., were sterilized with 5% (v/v) H_2_O_2_ for 5 min and rinsed with deionized water. The seeds were placed on filter paper soaked with deionized water in a tray and germinated in an incubator at 25 °C in the dark for 5 d. Subsequently, soybean seedlings with uniform sizes were selected, and each seedling was carefully planted in the mixed soil.

To initiate nodulation, a solution of rhizobia (*Sinorhizobium fredii*) was injected into each pot (1 mL, OD_600_ = 0.2). The seedlings were then placed in a greenhouse at the Chinese Agricultural University with a day/night cycle (16 h/8 h) and a temperature of 25/25 °C with a humidity of 70%. Each treatment sample was watered every 2 d with 150 mL of water. After 90 d, the water was changed to Hoagland nutrient solution to provide nutrients to the plants. Hoagland nutrient solution (Hopebiol, China) was prepared according to the manufacturer’s instructions. Briefly, 1.26 g of this product and 0.945 g of Ca(NO_3_)_2_ were dissolved in 1000 mL of deionized water and autoclaved at 115 °C for 30 min. The plants were harvested at different growth stages (30, 60, 90, and 115 d) for various end points and analyses.

At 30 d posttreatments (V6 stage), the seedlings were divided into shoots, roots, and nodules. The biomass and length of roots and shoots, several photosynthetic parameters, inorganic nutrient content, antioxidant activity, and metabolomic profile were measured to evaluate the plant response at the early growth stage. At day 115 (R8 stage, full maturity), soybean seeds were harvested to determine yield as well as organic and inorganic nutritional quality. The life cycle experiments were conducted autonomously in triplicate between 2019 and 2022. The resultant average yields from the three distinct experimental runs were subsequently employed to ascertain the overall mean values for the study. To determine the mechanism of action of MoS_2_ and the difference between the different Mo materials, Mo enzymes were quantified across the three key growing stages (V6, R3, and R6). The dynamic adsorption and biotransformation of MoS_2_ materials were determined by measuring the Mo and S content and chemical species in both plant tissues and soil using orthogonal techniques, including SP-ICP-MS and XANES.

### Elemental Analysis

Freeze-dried plant samples were ground into fine powders and digested in a mixture of nitric acid and hydrogen peroxide (v/v: 3:1) in a microwave digestion system (MARS 6, UK). Elements were then determined by ICP-MS (Thermo Scientific). Shoot tissues (GBW 07602) were used as standard reference materials as described by Zhang et al.^15^ Calibration standards of known concentrations (0.01–100 ppm) were used for quantification. The element recovery rates are presented in Supporting Information Table 3.

### SP-ICP-MS Analysis

SP-ICP-MS analysis was performed on MoS_2_ NP-treated plants. Fresh roots, shoots, and nodules collected at 30 d and seeds harvested at 115 d were digested using an enzymatic method described by Dan et al.^16^ Fresh samples were freeze-dried and ground to a fine powder in liquid nitrogen. Ten milligram samples were homogenized in 8 mL of 20 mM MES buffer (pH = 5), followed by the addition of 2 mL of macerozyme R-10 solution (25 mg/mL). The mixture was incubated for 24 h in an orbital shaker at 37 °C and 200 rpm. The mixture was left to stand for 30 min, and the supernatant was diluted 200 times for analysis by SP-ICP-MS (7700, Agilent Technologies, Palo Alto, CA). To examine whether the enzymatic process affects particle size, MoS_2_ NPs with a known amount were spiked with plant tissues and digested following the same procedure mentioned above. The size was then compared with that of a MoS_2_ NP suspension at the same concentration. For optimum instrument sensitivity, the tuning solutions (7Li, 59Co, 89Y, 205Tl, 140Ce, and 137Ba in 2% v/v HNO_3_ solution) provided by Agilent were used for analysis. The peristaltic pump sample uptake rate was 0.32 mL/min, the dwell time was set to 3 ms, and the sampling time was 60 s per sample (time-resolved mode, TRA). A suspension of 50 ng/L (60 nm) Au nanoparticles was used to determine the atomization efficiency. A standard solution of dissolved Mo (1 μg/L) was prepared in 1% nitric acid. The operating parameters of the instrument are shown in Supporting Information Table 4.

### Dissolution of MoS_2_

To understand the dissolution of MoS_2_ in soil with the presence of plants, soil pore water was collected at different time points over the course of 90 d. Sampling was performed using a soil pore water sampler (Eijkelkamp Agrisearch Equipment, The Netherlands) coupled with a 5 mL vacuum bottle. Soil pore water samples were collected every 15 d for analysis of Mo content using ICP-MS.

### Synchrotron Radiation XANES Analysis of Mo and S

Plant (roots, shoots, and nodules) and soil (rhizosphere soil and pot soil) samples were freeze-dried, ground into fine powder, and compressed into thin sheets for XANES analysis. Mo in samples was measured on beamline 1W1B of the Beijing Synchrotron Radiation Facility (BSRF) and beamline BL07A of the Taiwan Synchrotron Radiation Facility (TLS). The Mo K-edge (20,000 eV) spectra were collected using a 19-element Ge solid-state detector at the BSRF or a lytle solid-state detector at the TLS. Reference samples, including MoS_2_, MoCl_5_, Na_2_MoO_4_, Mo-malic acid, and Mo-cysteine were collected in transmission mode (Supporting Information Figure 3). Mo-malic acid and Mo-cysteine were synthesized using the protocol described by Zhou et al.^17^ and Kay and Mitchell,^18^ respectively. For molybdenum malic acid, a mixture of Na_2_MoO_4_ and sodium malic acid was prepared at a molar ratio of 1:2, with the pH adjusted to 5.0 using NaOH. The solution was heated in a water bath to 75 °C and maintained for 5 h. Then, the mixture was slowly evaporated at a 4 °C refrigerator and colorless crystals were precipitated after a few days. For Mo-cysteine, 64% l-cysteine hydrochloride and 60.8% Na_2_MoO_4_ were mixed in water. The solution of 25% sodium dithionite was then added. The mixture was vacuum-filtered, washed with 50% ethanol, and recrystallized three times from 50% ethanol to obtain Mo-cysteine.

The S K-edge (2472 eV) XANES spectra of plants and soil were collected on beamline 4B7A at the BSRF using a silicon drift detector in fluorescence mode. Reference samples, including MoS_2_, oxidized glutathione, reduced glutathione, Na_2_SO_4_, sodium dodecyl sulfate, sodium dodecyl benzenesulfonate (SDBS), and methionine sulfoxide (MS), were collected in total electron yield mode (Supporting Information Figure 4). Three spectra were collected for each sample for average. All of the XANES data were processed using Athena (0.9.26 version). Energy calibration and normalization of the spectra were done first and then analyzed by linear combination fitting (LCF) to calculate the ratio of different Mo or S chemical species.^19^Supporting Information Table 5 shows fitting parameters from LCF analysis of XANES spectra of Mo and S of samples.

### Statistical Analysis

The greenhouse experiment was a completely randomized design with six replicates of each treatment. Values are shown as the mean ± SD. Statistical analysis was performed on SPSS 19.0. Statistical significance was evaluated through one-way ANOVA. The mean values of each treatment were compared using Tukey’s test. *P* < 0.05 was significantly different.

## Results and Discussion

### Phytoeffects of MoS_2_ on Soybean

**Figure 1:**
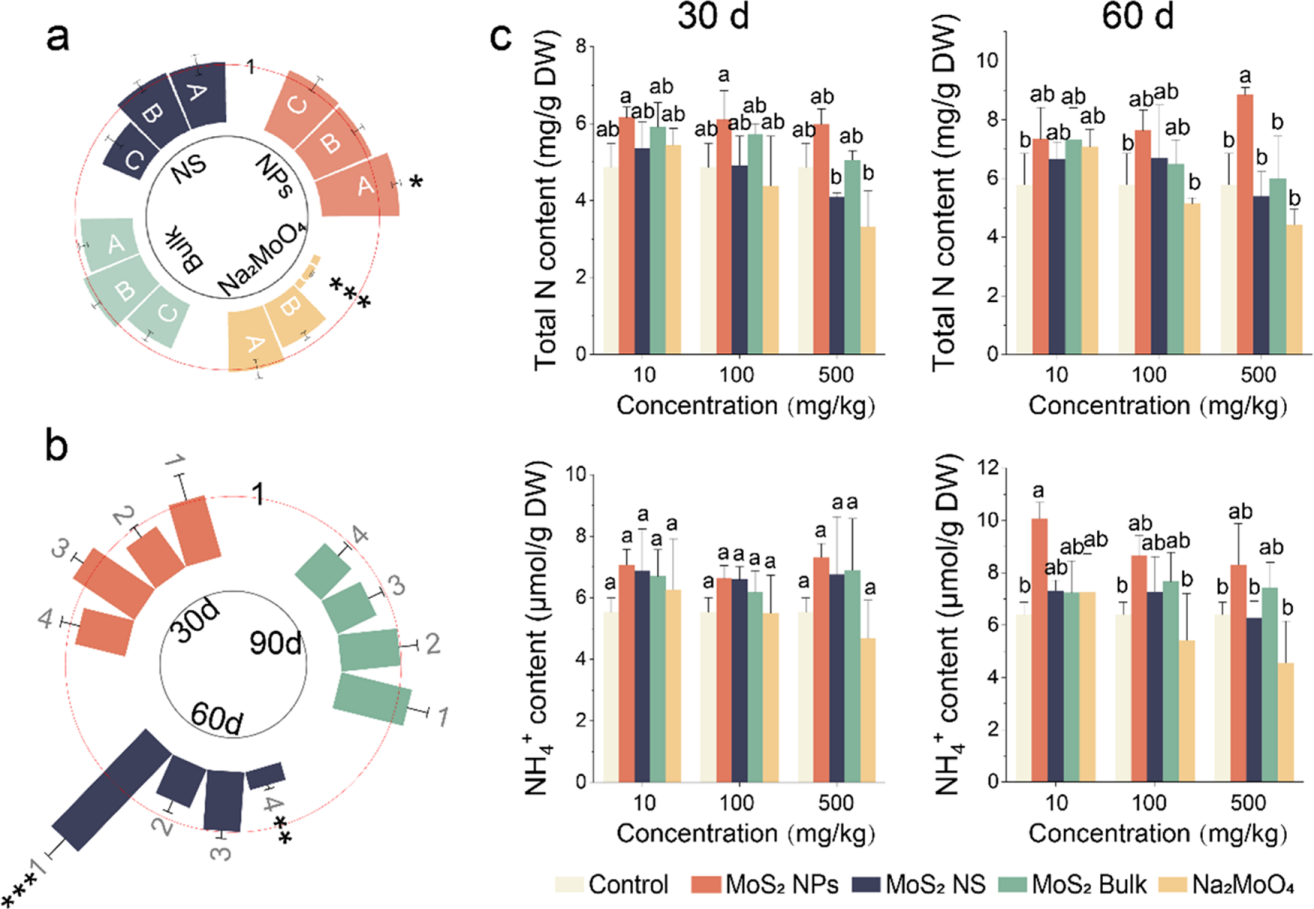
Soybean growth and nutrient analysis. (a) Yield of soybean grain (grain weight). A, B, and C represent 10, 100, and 500 mg/kg, respectively. (b) Nitrogenase activities in nodule at 60 d upon treatment with 500 mg/kg of the Mo-based materials. (c) Total nitrogen content and NH_4_^+^ content in nodules at 30 and 60 d. In panels (a) and (b), 1, 2, 3, and 4 represent treatments of 500 mg/kg MoS_2_ NPs, MoS_2_ NS, MoS_2_ bulk, and Na_2_MoO_4_, respectively. The height of the bar indicates the fold change relative to the control. *, **, and *** represent significant difference compared with control at *P* < 0.05, *P* < 0.01, and *P* < 0.001, respectively. In panel (c), different lowercase letters indicate significant differences between groups.

The four Mo-based materials show distinct effects on soybean yield. MoS_2_ NPs of 10 mg/kg increased the yield by 35% compared to the control and by 30% compared to the conventional molybdate fertilizer (Figure 1a and Supporting Information Table 6). There was no significant change in grain weight in MoS_2_ bulk and MoS_2_ NS treatments as compared to control. Therefore, MoS_2_ NP-treated soybean yield increased, which was superior to those of MoS_2_ bulk, MoS_2_ NS, and the conventional molybdenum fertilizer Na_2_MoO_4_ at 10 mg/kg. However, the other materials showed no positive effects on the yield, and Na_2_MoO_4_ inhibited the yield by 87% at 500 mg/kg (Figure 1a).

Supporting Information Figure 5 shows a heat map of the changes in nutrient content in soybean shoots and roots. There was no obvious disturbance in the homeostasis of the nutrient elements in roots and shoots after MoS_2_ NP (10, 100, and 500 mg/kg) treatments. In roots, 500 mg/kg MoS_2_ NS and Na_2_MoO_4_ disturbed elemental homeostasis, in which MoS_2_ NS significantly increased the Mn content and Na_2_MoO_4_ decreased the Fe content (Supporting Information Figure 5a). In shoots, MoS_2_ bulk disturbed Fe homeostasis and MoS_2_ NS and Na_2_MoO_4_ increased Mn at 500 mg/kg (Supporting Information Figure 5b). Plants require the nutrients Mn and Fe, but excessive amounts of these elements can be poisonous. Studies have shown that nanoparticle-induced acidification of the culture medium can cause iron overload, which negatively affects plant growth.^15,20^ The excessive Mn in shoots can inhibit photosynthesis by blocking the Fe-related chlorophyll synthesis process.^21^ These results agree with the yield results, implying that the effects of MoS_2_ are relevant to the amount of Mo ions since the molybdate with the highest amount of free Mo ions had the most significant negative impact.

MoS_2_ NS and MoS_2_ bulk treatments showed no significant difference in the effects on nitrogenase activities and nitrogen accumulation in nodules, while Na_2_MoO_4_ resulted in 70% reduction. The lack of changes in nitrate content may imply that the four materials have no direct effects on the other nitrogen metabolic pathways, such as nitrification (Supporting Information Figure 6). However, MoS_2_ NPs enhanced the nitrogenase activity by 122% even at a concentration of up to 500 mg/kg (Figure 1b). Correspondingly, MoS_2_ NPs (10, 100, and 500 mg/kg) increased the total nitrogen content in nodules by 27.0, 32.0, and 53.1% and NH_4_^+^ content by 57.4, 35.2, and 29.8%, respectively, at 60 d (Figure 1c). Therefore, the enhancement of nitrogenase activity by MoS_2_ NPs led to an increased total nitrogen accumulation, which is a crucial factor in boosting the soybean yield.

Our previous research revealed that MoS_2_ NPs increase nitrogenase activity for two reasons: first, MoS_2_ NPs release Mo ions essential for nitrogenase synthesis, and second, MoS_2_ NPs behave as nanoenzymes, scavenging ROS in nodules to shield nitrogenase from oxidative damage.^22^ The appropriate ratio of the 1T phase and lattice oxygen in MoS_2_ NPs provides them with exceptional catalytic properties, which present the potential to operate as the nanoenzyme in plants^23−25^ (Supporting Information Table 7). In addition, previous results indicated that MoS_2_ NPs have peroxidase (POD)-like and catalase (CAT)-like activities.^23^ However, the transformation of MoS_2_ NPs into Mo(VI) for nitrogenase synthesis and the maintenance of intact particles as nanoenzymes to scavenge ROS in nodules are seemingly a “paradoxical” process. Therefore, to elucidate this “paradoxical” process, we investigated the dynamic transformation of MoS_2_ NPs in a soil–soybean–nodule system.

### Biodistribution and Transformation of MoS_2_ in a Soil–Plant System

**Figure 2:**
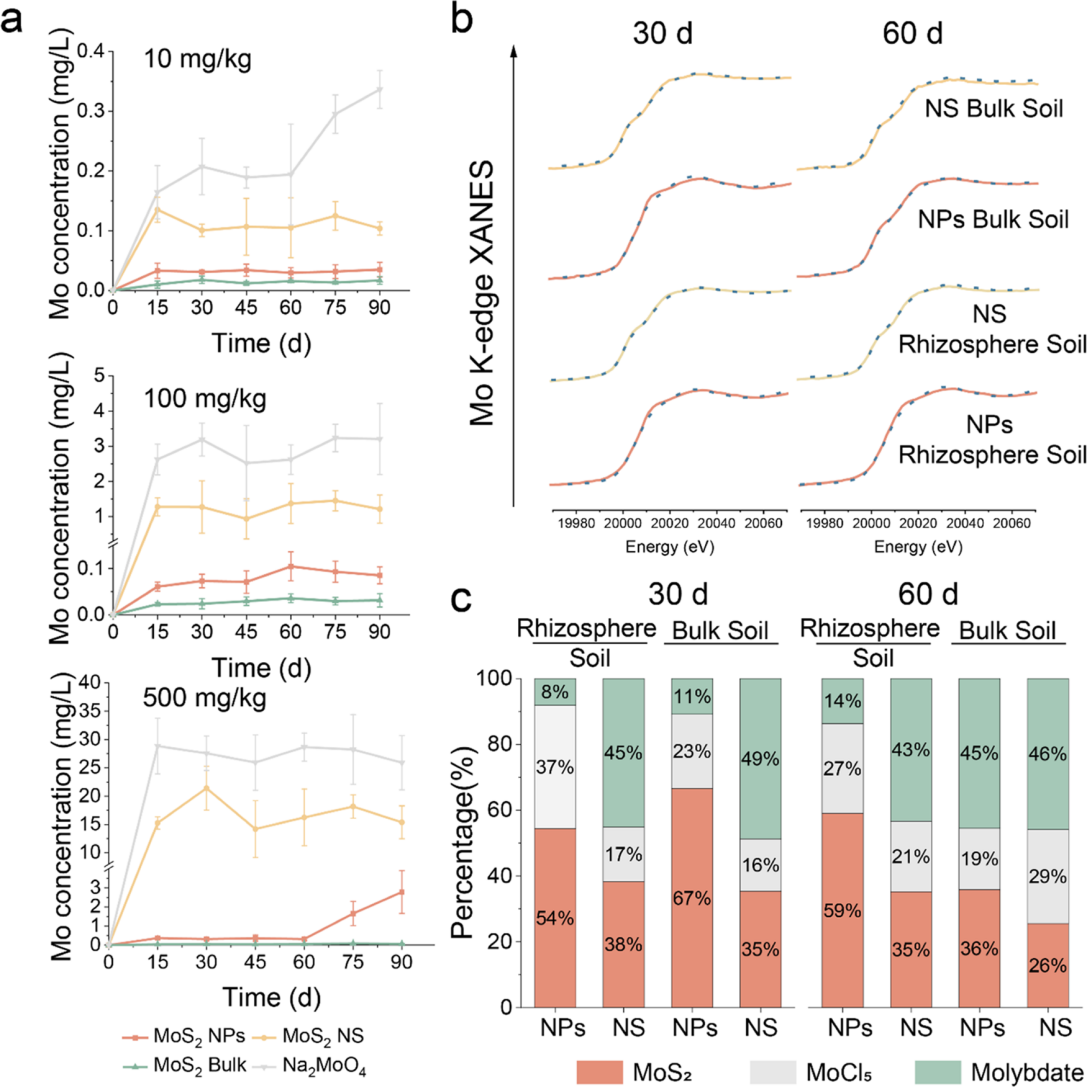
Transformation of MoS_2_ in soil. (a) The content of free Mo in soil pore water after incubation of the four materials at 10 mg/kg, 100 mg/kg, and 500 mg/kg for 15, 30, 45, 60, 75, and 90 d in soil. (b) Mo K-edge XANES spectra of pot soil and rhizosphere soil samples. The solid line indicates original spectra and the dotted line indicates fitted lines generated from linear combination fitting (LCF) analysis. Concentration of the treatments is 500 mg/kg. (c) Fractions of Mo species were obtained by LCF analysis of the spectra.

We first investigated the dissolution of the Mo-based materials in soil by measuring free Mo content in soil pore water and dissolved Mo percentage in deionized (DI) water, root exudates, and soil leachate. The content of the released Mo from different materials was in the order of Na_2_MoO_4_ > MoS_2_ NS > MoS_2_ NPs > MoS_2_ Bulk (Figure 2a and Supporting Information Figure 7). The MoS_2_ NS exhibited a higher dissolution rate than did MoS_2_ NPs and MoS_2_ bulk. The highest 2H phase ratio and bulk-like morphology make MoS_2_ bulk nearly insoluble (Supporting Information Figure 1 and Table 7). We next examined the transformation using XANES, comparing MoS_2_ NPs and MoS_2_ NS given their differences in dissolution and phytoeffects.

As shown in Figure 2b,c, Mo was presented as three chemical species, i.e., MoS_2_, MoCl_5_, and molybdate, demonstrating the transformation of MoS_2_ in soil. MoS_2_ NS exhibited a more significant and faster transformation compared to MoS_2_ NPs, particularly in rhizosphere soils. MoS_2_ bulk was virtually insoluble. This trend is consistent with dissolution in soil pore water. Over 60% of the MoS_2_ NS had already transformed to MoCl_5_ and molybdate at 30 d, while for MoS_2_ NPs, this was only 33–45%. Nanomaterial dissolution and transformation are highly affected by their physicochemical properties.^26^ Materials with a greater surface area show higher ability for adsorbing organic ligands within the soil, forming an aggregation barrier which accelerates their dissolution.^27^ Compared to the 2H phase, the 1T phase of MoS_2_ exhibits higher solubility and oxidation rates, demonstrating greater environmental reactivity.^28^ The MoS_2_ NS exhibits the highest proportions of the 1T phase, lattice oxygen, and surface area, with values of 1, 34.8%, and 12.87, respectively, whereas MoS_2_ NPs show lower values of 0.71, 10.9, and 9.29%. This results in a higher transformation rate for MoS_2_ NS compared to MoS_2_ NPs (Supporting Information Figures 1 and 2 and Table 7).

The fractions did not undergo obvious changes in rhizosphere soil over time, while for MoS_2_ NPs in pot soil, a gradual transformation from 30 to 60 d could be observed. This suggested that root exudates can accelerate the transformation processes and lead to a fast transformation near the root surface in the initial 30 d. While in pot soil, which was away from the root, MoS_2_ NPs transform gradually over time. The ratio of 1T and 2H phases and the surface area of MoS_2_ NPs allow them to dissolve and transform at an appropriate speed that is best for enhancing BNF and plant growth (Supporting Information Figures 1 and 2, Table 7). This slower process allowed MoS_2_ NPs to remain intact particles and perform their nanozyme function in the soil around the root/nodule while releasing Mo in a more sustainable way.

**Figure 3:**
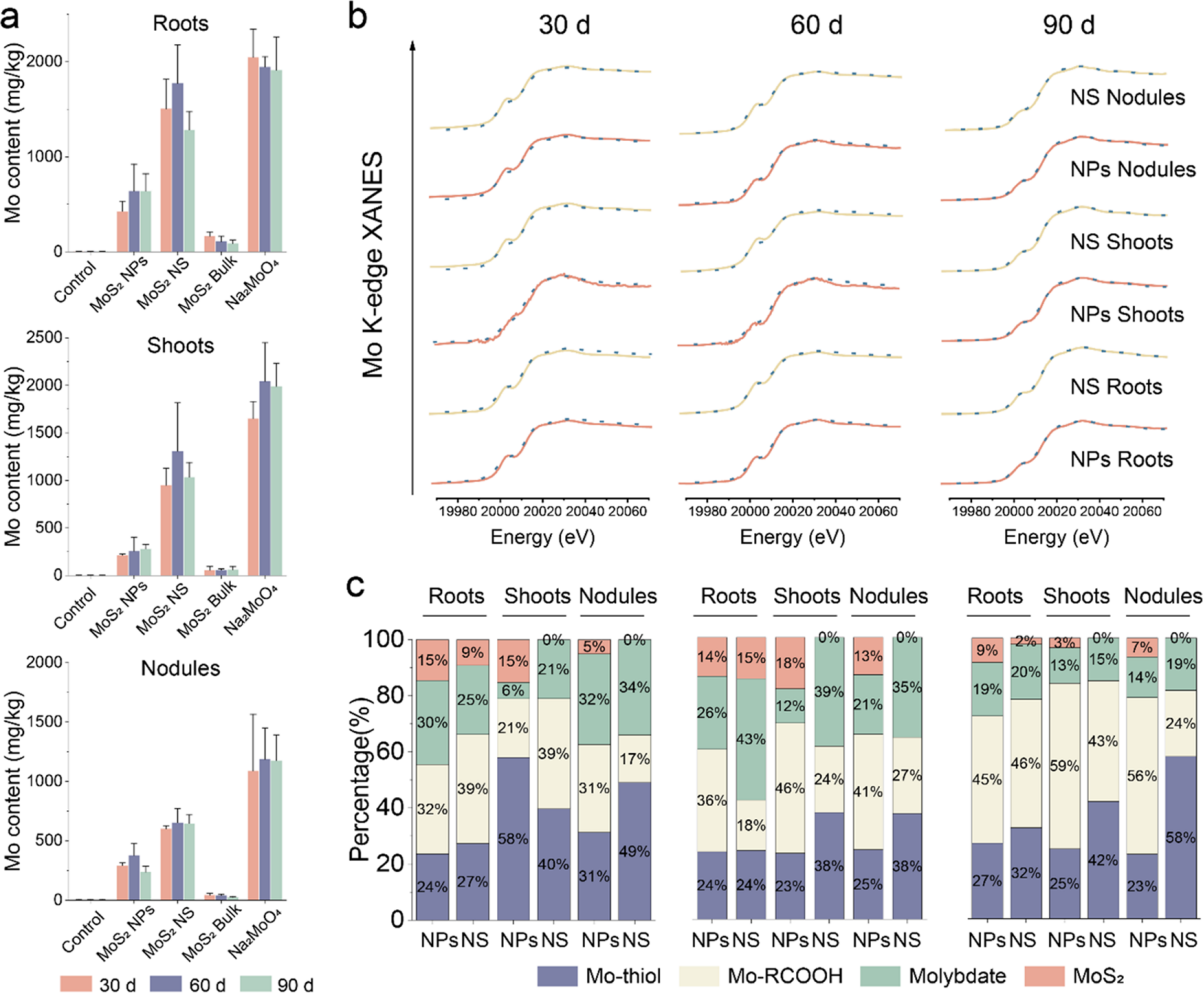
Biotransformation of MoS_2_ in soybean. (a) Mo content of soybean roots, shoots, and nodules treated with 500 mg/kg of four materials and control group at 30, 60, and 90 d. (b) Mo K-edge XANES spectra of soybean samples. The solid line indicates original spectra and the dotted line indicates fitted lines generated from linear combination fitting (LCF) analysis. Concentration of the treatments is 500 mg/kg. (c) Fractions of Mo species obtained by LCF analysis of the spectra.

We then examined the biodistribution and chemical species of Mo in plant tissues. Na_2_MoO_4_ and MoS_2_ NS-treated soybeans had the highest Mo content in all tissues, ranging from 108.7 to 193.5 mg/kg at 10 mg/kg and 401.9 to 1368.7 mg/kg at 100 mg/kg, followed by MoS_2_ NPs at 10 mg/kg, with tissue Mo contents ranging from 71.9 to 117.7 mg/kg and 91.8 to 263.3 mg/kg in the tissue at 100 mg/kg. The Mo content in soybean roots treated with 500 mg/kg MoS_2_ NPs was 426.2, 641.6, and 640.5 mg/kg at 30, 60, and 90 d, respectively (Supporting Information Figure 8). The Mo content in soybean roots treated with MoS_2_ NS and Na_2_MoO_4_ was 1.0–2.5 and 2.0–3.8 times higher than that of the MoS_2_ NP treatment, respectively. The Mo content in 500 mg/kg MoS_2_ NP-treated soybean shoots was 213.1, 257.6, and 279.8 mg/kg. The Mo content in shoots treated with MoS_2_ NS and Na_2_MoO_4_ was 2.7–4.1 and 6.1–6.9 times higher than that of the MoS_2_ NP treatment, respectively. In most agricultural conditions, plants are tolerant to Mo toxicity. Some plants can tolerate up to several thousands of ppm of Mo in their tissues without detrimental growth effects.^29,30^ In our study, soybeans can tolerate 640 mg/kg of Mo in their roots (500 mg/kg MoS_2_ NP treatment); however, toxicity occurred in MoS_2_ NS and Na_2_MoO_4_ treatments, which lead to over 1000 mg/kg in shoots and roots. The Mo content in soybean nodules treated with MoS_2_ NPs was 292.5, 377.6, and 238.2 mg/kg at 30, 60, and 90 d, respectively (Figure 3a). The Mo content in soybean nodules treated with MoS_2_ NS and Na_2_MoO_4_ was 0.7–1.7 and 2.1–3.9 times higher than that of MoS_2_ NP treatment, respectively (Figure 3a). This corresponds to the results of the transformation rate and dissolution of the material (Figure 2). Soybeans treated with 500 mg/kg MoS_2_ NS and Na_2_MoO_4_ exhibited a significantly elevated Mo content compared with other treatments, leading to the inhibition of soybean growth. However, MoS_2_ NP treatments increased the Mo content to an appropriate amount, providing a substantial Mo source for the synthesis of Mo enzymes, including nitrogenase. This facilitates nitrogen assimilation in plants, a factor deemed indispensable for the enhancement of soybean yield. These results suggest that MoS_2_ NPs resulted in positive effects on the yield with less Mo accumulation in the plant, demonstrating that the MoS_2_ NPs are not only more beneficial but also safer for application than the conventional molybdate fertilizer.

XANES results suggested that MoS_2_ NPs can be adsorbed by roots and nodules and translocated to the shoots, whereas the transfer of MoS_2_ NS is far more limited. MoS_2_ NPs were detected at all time points in all tissues, comprising 9–15% of all Mo forms in roots, 3–18% in shoots, and 5–13% in nodules. In contrast, the detection of MoS_2_ NS was limited to the roots, accounting for 2–15% of all Mo forms (Figure 3c). The presence of MoS_2_ NPs in all tissues of soybean provides a prerequisite for MoS_2_ NPs to function as nanozymes, while MoS_2_ NS was only present in the roots unable to transfer to the nodules or shoots, which limited the nanozyme function of MoS_2_ NS.

Molybdate is the primary form of plant uptake and utilization from soil. The presence of molybdate form in the plant samples suggests that MoS_2_ was transformed and released molybdate for plant nutrition. There might be two sources of molybdate, including direct root uptake from the soil (transformed from MoS_2_ in soil) and the direct transformation of MoS_2_ NPs inside plant tissues. Indeed, in-plant transformation of nanomaterials has been demonstrated previously.^31^ Molybdate in plants can undergo further transformation into organic forms, including Mo-COOH (organic acid-bound Mo, including Mo-malate and Mo-citrate, etc.) and Mo-thiol (thiol-bound Mo, including Mo-cysteine and Mo-GSH, etc.) in soybean (Figure 3b). These two forms of Mo can be generated in different plant compartments, including the xylem and phloem walls and cell walls (which are abundant in carboxyl groups), as well as in the cytosol, during the translocation of molybdate. Thiols and organic acids are common biomolecules involved in the solubilization and release of NMs in plants.^32,33^ Therefore, organic acids and thiols probably induced the dissolution of MoS_2_ NPs in soybean tissues and chelated them with the dissolved molybdate to form Mo-RCOOH and Mo-thiol. It is worth noting that Mo-thiol and Mo-RCOOH are crucial structures for the iron–molybdenum cofactor (FeMoco, a cofactor of nitrogenase) and the molybdopterin–molybdenum cofactor (MPT/Moco, cofactor of XDH, AO, and NR). Organic acids are also essential for the synthesis of nitrogenase in nodules, which are Mo cofactors of nitrogenase formed by the combination of iron–sulfur clusters, Mo, and high citric acid.^34^

Indeed, the activities of XDH, AO, and NR were affected, with MoS_2_ NPs showing positive effects (Supporting Information Figure 9). AO activity was enhanced by 84 and 64% at 30 and 60 d, respectively, after MoS_2_ NP treatment. NR and XDH were also upregulated by MoS_2_ NPs, while the XDH activity at 90 d was inhibited by Na_2_MoO_4_ treatments. These results suggest that MoS_2_ transforms in both the soil and plant and can result in the release of molybdate as a nutrient, which can be further incorporated into nitrogenase and Mo enzymes.

**Figure 4:**
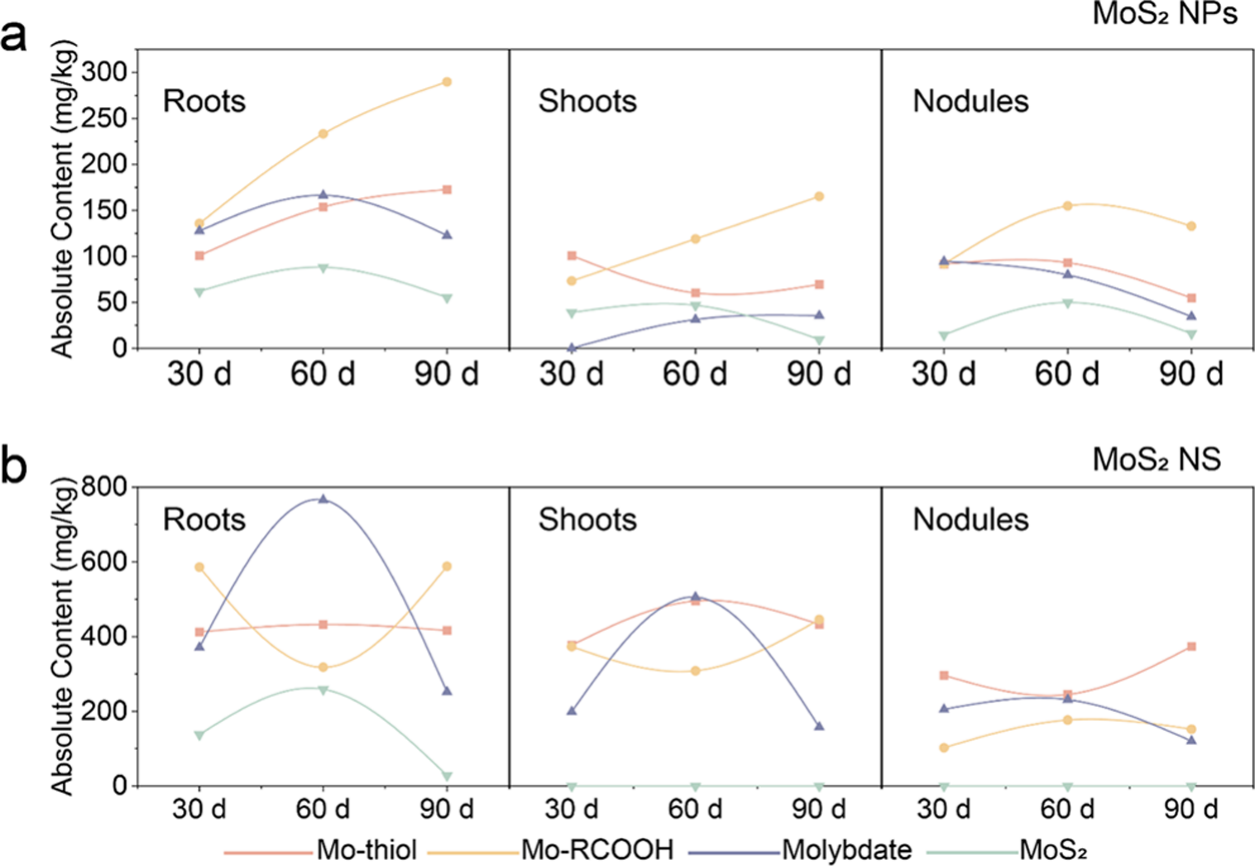
Mo species analysis. (a, b) Absolute content change of Mo species in roots, shoots, and nodules treated with 500 mg/kg MoS_2_ NPs and MoS_2_ NS.

To further understand the dynamic transformation process, we calculated the absolute amounts of the different Mo species by multiplying their fractions by the total Mo content (Figure 4). Overall, the change curve of Mo species absolute content in soybean treated with MoS_2_ NPs remained relatively stable, while those treated with MoS_2_ NS showed greater volatility. For MoS_2_ NP treatment (Figure 4a), the absolute contents of MoS_2_ species at 30, 60, and 90 d were 62.0, 88.2, and 55.3 mg/kg in roots, 39.0, 46.9, and 9.5 mg/kg in shoots, and 14.7, 49.9, and 16.0 mg/kg in nodules, respectively. The absolute contents of MoS_2_ NPs and soluble Mo (molybdate Mo-RCOOH and Mo-thiol) remained elevated at 30 and 60 d and decreased at 90 d. However, more dramatic changes were evident with MoS_2_ NS at 60 d, the most significant of which was a rapid increase in molybdate content in the roots and shoots by 1.07 and 1.54 times, corresponding with a similarly rapid reduction in Mo-COOH content by 0.46 and 0.17 times, respectively, compared with 30 d (Figure 4b). This phenomenon may be attributable to plant-derived carboxyl groups associated with a large quantity of rapidly dissolved Mo from the MoS_2_ NS, thereby leading to a sudden increase in free molybdates. In contrast, MoS_2_ NPs were readily adsorbed and translocated in plants, as evidenced by the steady increase in the total Mo content and Mo species in tissues. This implied that MoS_2_ NPs persist as intact particles in soybean tissues during early growth stages, thereby functioning as nanozymes that capture ROS for nitrogenase protection. Moreover, they continuously dissolve to release additional Mo that serves as a feedstock for Mo enzyme synthesis. Conversely, the high transformation rate of MoS_2_ NS resembles that of Na_2_MoO_4_, which has no positive effect or leads to toxicity at high doses. In short, the transformation rate of MoS_2_ NPs seems more biologically appropriate, resulting in sufficient Mo accumulation to support enhanced Mo enzyme functions, BNF, and plant growth.

**Figure 5:**
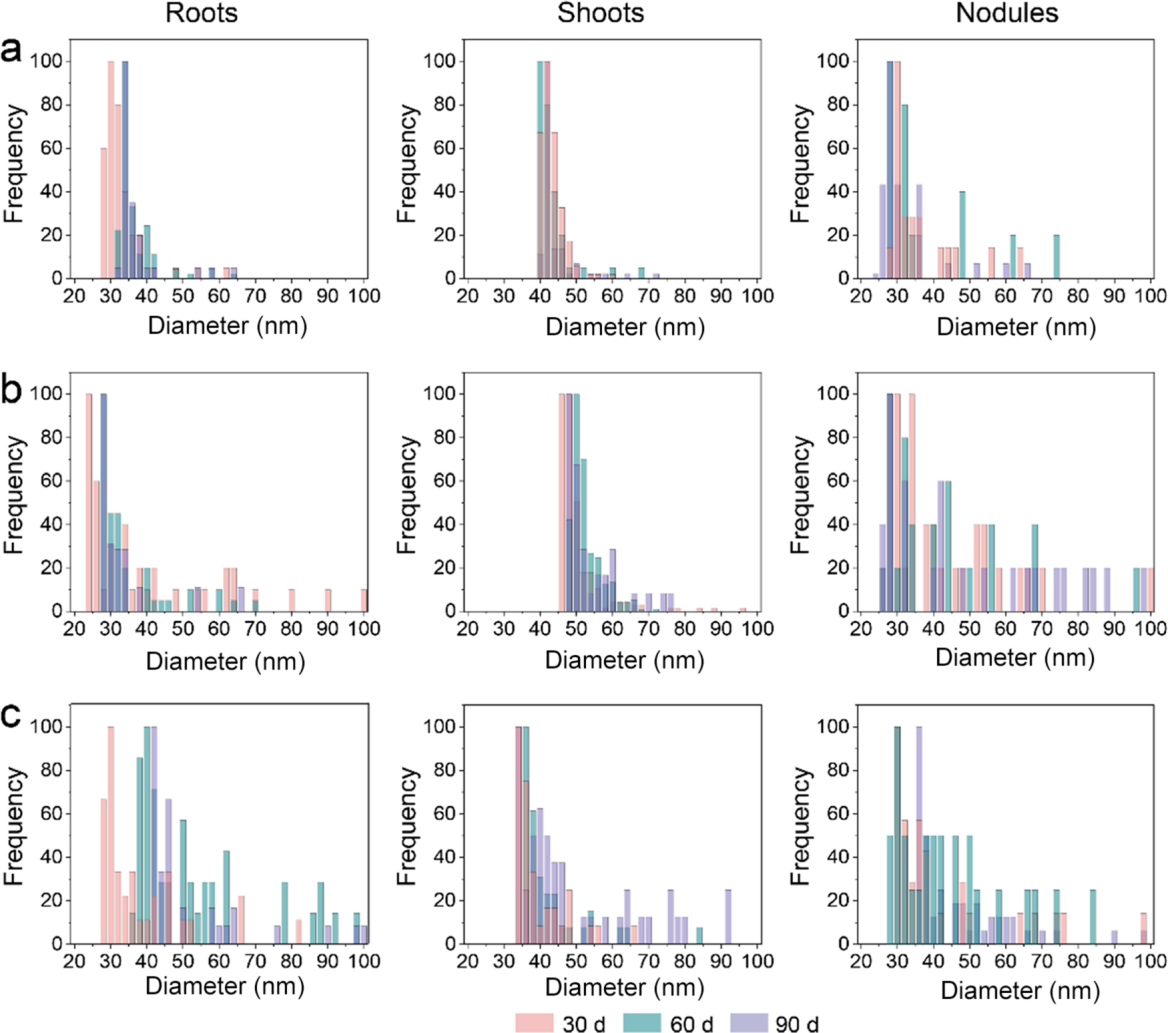
Particle size distribution histograms of soybean roots, shoots, and nodules after treatment with 10 mg/kg (a), 100 mg/kg (b), and 500 mg/kg (c) MoS_2_ NPs in 30, 60, and 90 d determined by SP-ICP-MS.

Since the presence of intact MoS_2_ NPs is critical for nanozyme function, we further analyzed the particle size in plant tissues using SP-ICP-MS. Only the MoS_2_ NPs were evaluated due to their spherical shape, unlike the flake-like MoS_2_ NS, as SP-ICP-MS assumes spherical particles for calculation and measurement. Preliminary experiments confirmed that macerozyme R-10 did not affect the particle size or concentration of MoS_2_ NPs (Supporting Information Figure 11). Particle size distribution of MoS_2_ NPs exhibited temporal variation in different plant tissues. Specifically, the average particle size of the MoS_2_ NPs ranged from 32.7 to 52.6 nm in roots, 42.5 to 53.8 nm in shoots, and 32.2 to 50.2 nm in nodules, with secondary peaks appearing in both roots and nodules (Figure 5 and Supporting Information Table 8). The average particle size was smaller than the initial pristine size of the suspension (average size: 85 nm) (Supporting Information Figure 11), suggesting that these smaller particles were generated from the transformation of MoS_2_ NPs, which may have subsequently improved their ability to cross the plant barrier. The changes in particle size in roots, shoots, and nodules, along with the emergence of secondary peaks in roots, provide direct evidence for the transformation of MoS_2_ NPs in plants.

**Figure 6:**
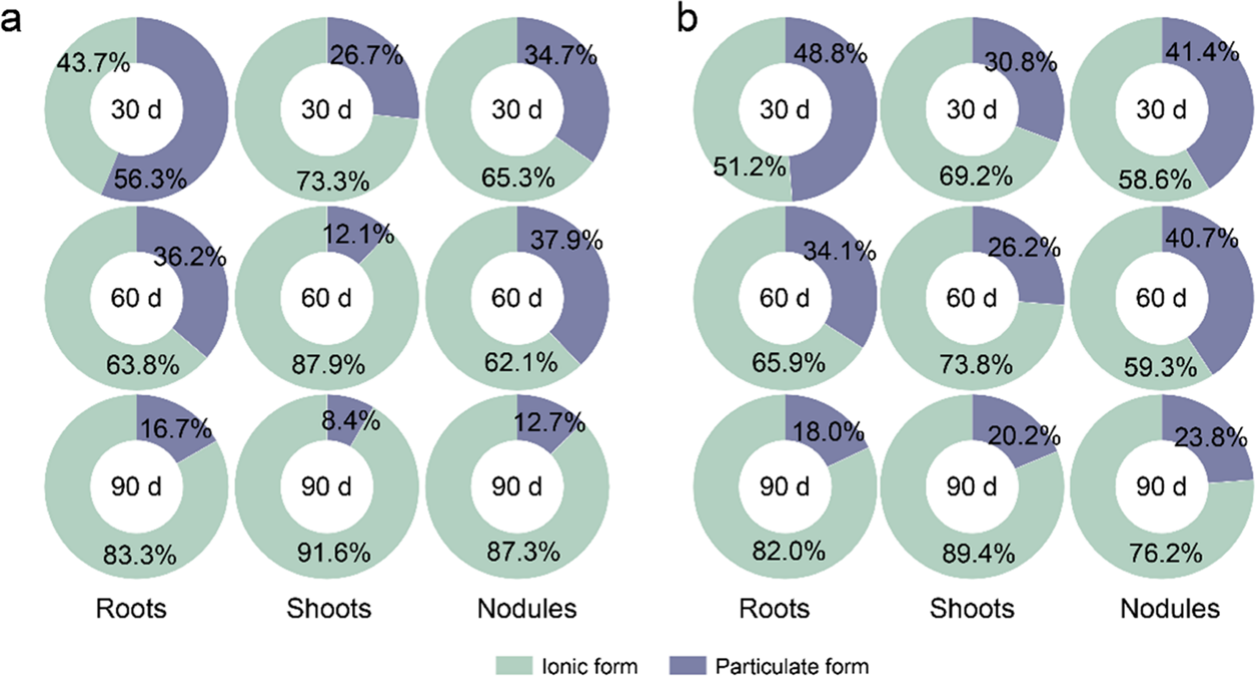
Form composition (ionic form and particulate form) of MoS_2_ in tissues of soybean treated by MoS_2_ NPs at 10 (a) and 100 mg/kg (b).

The proportion of the particulate form in soybean tissues treated with MoS_2_ NPs gradually decreased. Specifically, the particulate MoS_2_ in tissues treated with 10 and 100 mg/kg MoS_2_ NPs, respectively, decreased from 56.3 to 16.7% and 48.8 to 18.0% in roots and from 26.7 to 8.4% and 30.8 to 20.2% in shoots, while in the nodules, a reduction was observed from 34.7 to 12.7% and 41.4 to 23.8%, respectively (Figure 6). In addition, the higher proportion of particulate MoS_2_ in nodules relative to that in shoots can be likely ascribed to the pathway of MoS_2_ NP translocation (Figure 6). Upon initial absorption by the roots, MoS_2_ NPs accumulate in the adjacent nodules, a process that potentially facilitates their ROS-scavenging function for nitrogenase protection, while their translocation to shoots via the xylem faces increased physiological and physical constraints. These findings further substantiated that MoS_2_ NPs initially exist in plants in a particulate form functioning as nanoenzymes and subsequently dissolve and transform gradually to support Mo enzyme synthesis, which synergistically promoted nitrogen assimilation for improved soybean production.

### Sulfur Biodistribution and Transformation

**Figure 7:**
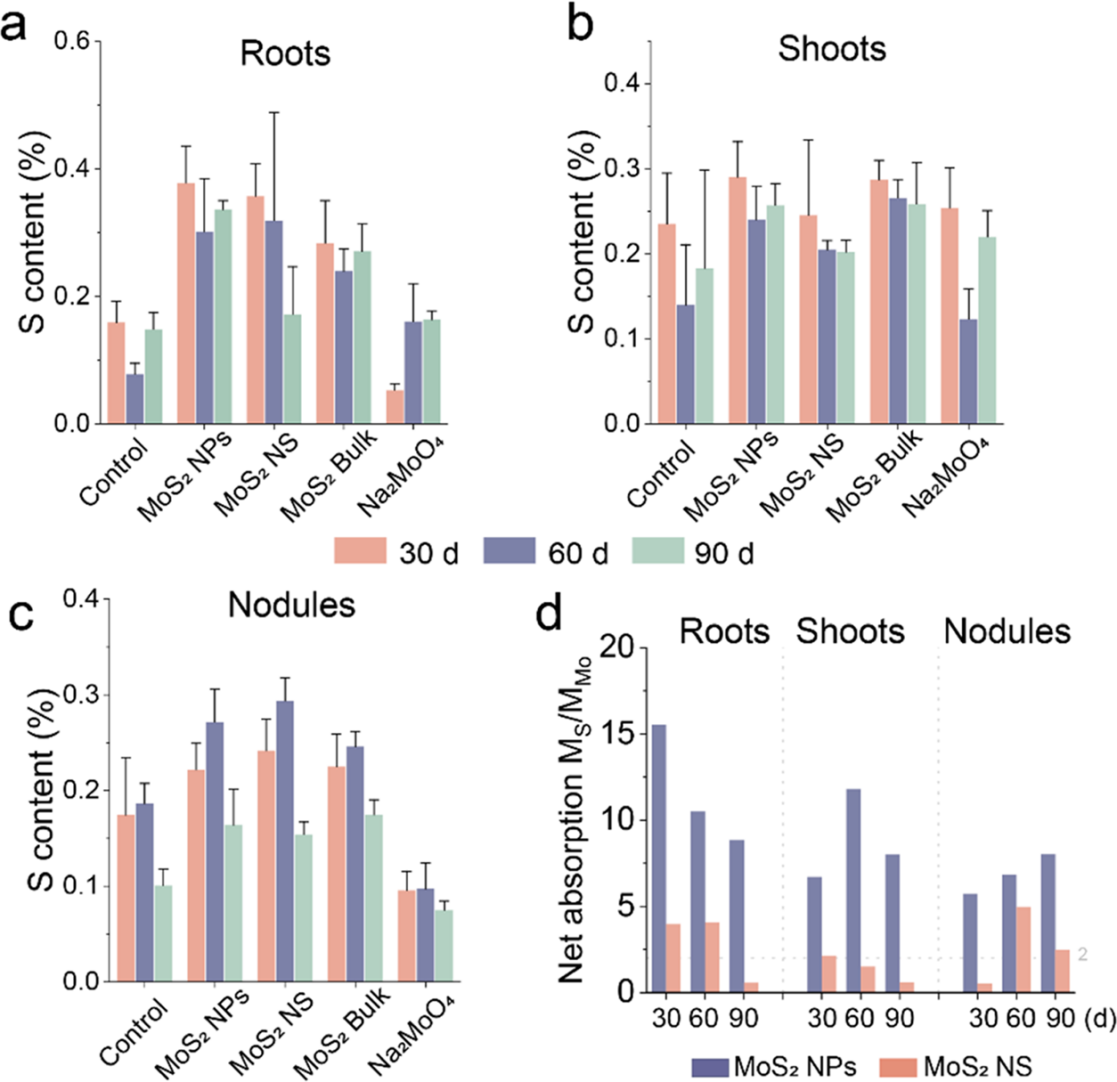
Biodistribution of S in soybean. (a–c) S content of soybean roots, shoots, and nodules treated with 500 mg/kg of four materials and control group at 30, 60, and 90 d. (d) Net absorption M_S_/M_Mo_ in soybean treated by 500 mg/kg MoS_2_ NPs and MoS_2_ NS at 30, 60, and 90 d.

Another key feature of MoS_2_ is the sulfur component, which is a macronutrient essential for plant growth. Therefore, it is hypothesized that the sulfate released during oxidative dissolution can also be absorbed by plants and incorporated into the synthesis of sulfur-containing biomolecules, such as proteins and antioxidants like glutathione (GSH). To investigate this hypothesis, the uptake of S by soybeans was measured. The total sulfur content of MoS_2_ NPs and MoS_2_ NS-treated plants increased in roots (137 and 125%) and nodules (27 and 38%) at 30 d, respectively, but was reduced by 67% in roots and 57% in nodules after Na_2_MoO_4_ treatment (Figure 7a–c). This reduction of sulfur content with Na_2_MoO_4_ could be due to the rapid release of a large pool of molybdate, which can bind sulfur and reduce uptake or compete with sulfate for transport channels in plants; this effect would decrease over time as the molybdate is absorbed by the plant.

Interestingly, the dissolution rate of MoS_2_ NPs was actually lower than the NS as shown above (Figure 2a and Supporting Information Figure 7) but actually resulted in greater in-planta sulfur content, suggesting the involvement of other mechanisms. Since far greater amounts of MoS_2_ NPs accumulated in the plant than did MoS_2_ NS, it appears likely that a significant amount of the sulfate release occurred in MoS_2_ NP-treated plants. To confirm this view, the ratio between the net molar concentrations of Mo and S uptake by plants was used to characterize the disparity in plant uptake of these two elements (Figure 7d). Specifically, the net absorption of M_S_/M_Mo_ was obtained by dividing the net increase of S by the net increase of Mo. The M_S_/M_Mo_ values in soybean roots treated with MoS_2_ NPs were 15.52, 10.49, and 8.83, while those treated with MoS_2_ NS were 3.95, 4.07, and 0.54 at 30, 60, and 90 d, respectively. The higher M_S_/M_Mo_ values were observed in the MoS_2_ NP treatment compared to the MoS_2_ NS treatment, suggesting a greater net adsorption ratio of S/Mo in the MoS_2_ NP-treated plants. Overall, the gradual increase in plant S content until 90 d, coupled with the significantly greater net adsorption ratio of S/Mo, supported the above view.

**Figure 8:**
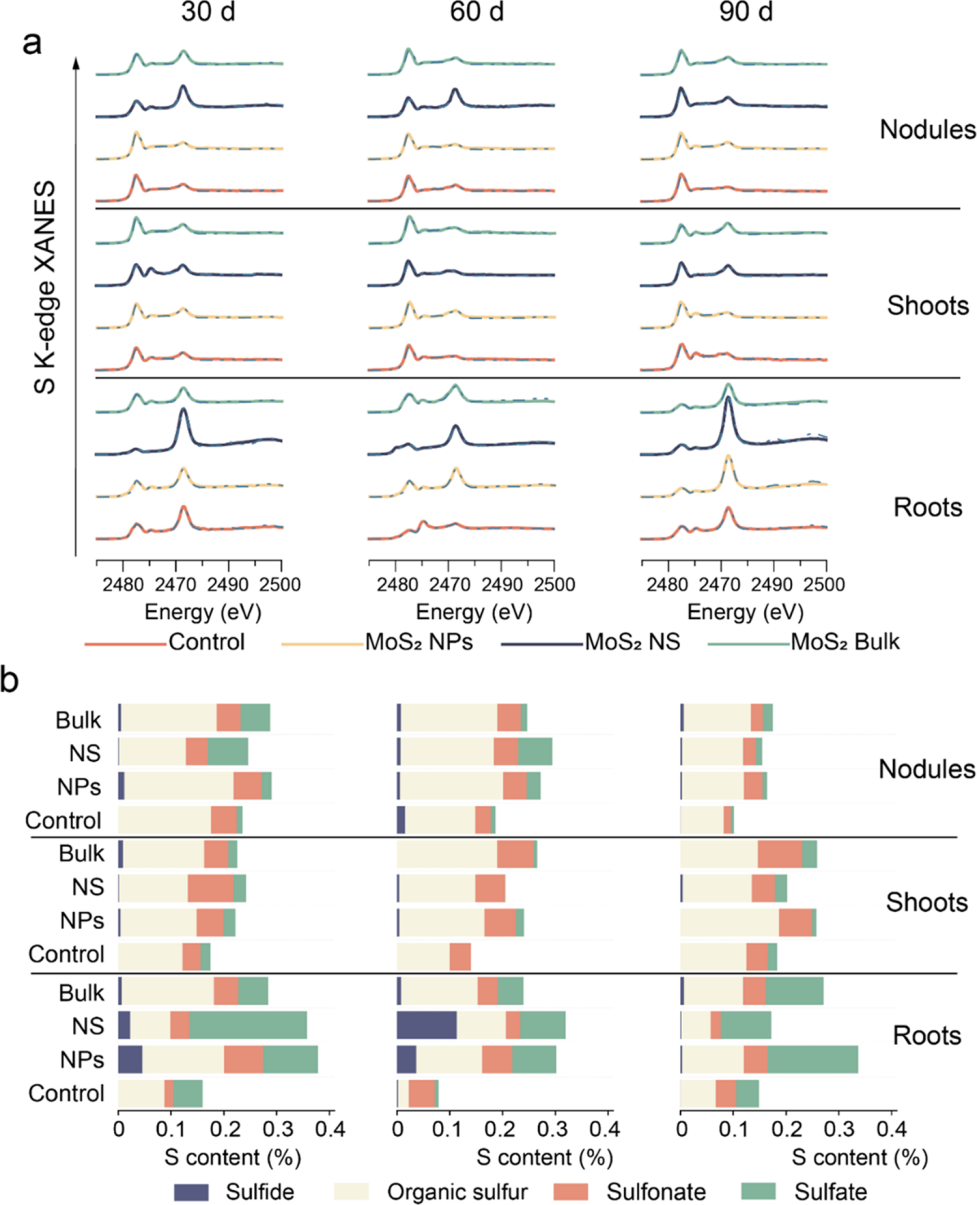
Biotransformation of S in soybean. (a) S K-edge XANES spectra of soybean samples. The solid line indicates original spectra and the dotted line indicates fitted lines generated from linear combination fitting (LCF) analysis. Concentration of the treatments is 500 mg/kg. (b) Absolute content of the S species obtained by LCF analysis of the spectra. In panel (b), the length of the column represents the S content expressed as the percentage of S in total tissue biomass (w/w).

Sulfur is a structural component of key proteins, vitamins, and cofactors, including the Moco and FeMoco.^22,35^ Plants largely accumulate sulfate from soil and readily assimilate this into other species, while they can also absorb and use sulfur in different forms using different assimilation pathways.^36^ Based on the structure, we classified the in-plant sulfur into four groups, sulfide (MoS_2_), thiol compounds (R-SH), sulfoxide (R-SO or R-SO_2_), and sulfate (SO_4_^2–^) and used them for analyzing the S species by XANES (Figure 8a). The absolute content of S species was calculated by multiplying the total S content by the fractions of S species and expressed as the percentage of the total plant biomass (Figure 8b). Results suggest that sulfur mainly exists as thiol compounds in the plants, followed by sulfoxide and sulfate.

Thiol compounds play critical roles in antioxidant and detoxification processes as well as nodule function. The control group exhibited a significant decrease in thiol content in the nodules after 60 d, as tissues began to age, indicating self-defense weakened in plants.^37^ MoS_2_ NP treatment increased the content of thiols by 77, 522, and 75% at 30, 60, and 90 d in roots, respectively, as compared with controls (Figure 8b). This increase was particularly significant at 60 d (by 522%) when the efficiency of BNF was the highest. This elevated thiol content can protect nodule tissues from the ROS damage associated with aging. This likely contributed to the high BNF efficiency (the high nitrogenase activity) that was maintained even at 60 d, which is a time point when nodule aging typically occurs and leads to declining BNF.^38^ Conversely, MoS_2_ NS treatments increased the contents of sulfate in all plant tissues, with 3.07, 12.14, and 1.22 times increase in roots and with 6.46, 6.43, and 1.40 times increase in nodules at 30, 60, and 90 d compared with the control, respectively. Excessive sulfate can be harmful to plant growth. Early and rapid accumulation of large amounts of sulfate in the roots and nodules can dysregulate normal sulfate assimilation and cause cytotoxicity.^39^ Therefore, it causes the production of large amounts of reactive oxygen species and prevents the synthesis of thiols, which may accelerate the senescence of nodules.^40^ This was further demonstrated by the reduced MDA content in the MoS_2_ NP-treated nodule samples (Supporting Information Figure 12).

### Environmental Implications

The results of this research have significant implications for the environment, especially in the context of sustainable agricultural practices. Our investigation into MoS_2_ and its interactions within the soil–plant system offer insights into both the potential benefits and challenges of utilizing nanomaterials in agriculture. The discovery suggests that nano-MoS_2_ has the potential to enhance biological nitrogen fixation, which could lead to more effective nutrient management in agriculture. However, the excessive transformation of MoS_2_ NS resulted in the overaccumulation of Mo within plants, detrimentally affecting nodule function and crop yield. This outcome underscores the importance of managing the application of nanomaterials to prevent unintended ecological risks. The study thus highlights the importance of understanding nanomaterial transformation when designing nanoagrochemicals thereby harnessing the advantages of nanomaterials while minimizing potential adverse effects. Future research in this field should also concentrate on refining application methods and dosages to ensure the responsible and efficient use of nanomaterials in agriculture, safeguarding the environment, and long-term food production.

## Supporting Information Available

Details of the experimental methods: nanomaterial and soil characterization measurement: MoS_2_ characterization, Mo and S K-edge XANES of reference materials, nutrient levels in roots and shoots, MoS_2_ dissolution characteristics, Mo content in soybean tissues (10 and 100 mg/kg), effects of materials on Mo enzymes, particle size distribution of MoS_2_ NPs and MDA content in nodules. Supporting Information Table: Soil parameters, hydrodynamic size and ζ potential of materials, ICP-MS detection parameter, SP-ICP-MS instrument parameters, fitting parameters from LCF analysis, soybean grain yield, XPS fitting results, and average MoS_2_ NP size in soybean tissues (PDF)
