# Supplementary material for: Dynamic Transformation of Nano-MoS2 in a Soil–Plant System Empowers Its Multifunctionality on Soybean Growth
Source: Environ Sci Technol. 2024 Jan 4;58(2):1211–22. doi: 10.1021/acs.est.3c09004 (PMC10795185; doi:10.1021/acs.est.3c09004)
Supplement: Supplementary file 1 — es3c09004_si_001.pdf [file es3c09004_si_001.pdf]

Supporting Information

Dynamic transformation of nano-MoS_2 in soil-plant system empower its multifunctionality on soybean growth

Mingshu Li[1,2,3†], Peng Zhang[1,4,†,*], Zhiling Guo[4], Weichen Zhao[5], Yuanbo Li[2], Tianjing Yi[2], Weidong Cao[6], Li Gao[7,*], Chang Fu Tian[8], Qing Chen[2], Fazheng Ren[9], Yukui Rui[2,*], Jason C. White[10,*], Iseult Lynch[4]

[1]Department of Environmental Science and Engineering, University of Science and Technology of China, Hefei 230026, China

[2]College of Resources and Environmental Sciences, China Agricultural University, Beijing 100193, China

[3]China CDC Key Laboratory of Environment and Population Health, National Institute of Environmental Health, Chinese Center for Disease Control and Prevention, Beijing 100021, China

[4]School of Geography, Earth and Environmental Sciences, University of Birmingham, Edgbaston, Birmingham, B15 2TT, UK

[5]State Key Laboratory of Environmental Chemistry and Ecotoxicology, Research Center for Eco-Environmental Sciences, Chinese Academy of Sciences, Beijing 100085, China

[6]Institute of Agricultural Resources and Regional Planning, Chinese Academy of Agricultural Sciences, Beijing 100081, China

[7]State Key Laboratory for Biology of Plant Disease and Insect Pests, Institute of Plant Protection, Chinese Academy of Agricultural Sciences, Beijing 100193, China

[8]State Key Laboratory of Agrobiotechnology, College of Biological Sciences, China Agricultural University, Beijing 100193, China

[9]Key Laboratory of Precision Nutrition and Food Quality, China Agricultural University, Beijing 100083, China

[10]The Connecticut Agricultural Experiment Station, New Haven, CT 06504, United States

† These authors contributed equally.

*Corresponding authors: jackyzhan1987@gmail.com (P.Z.); ruiyukui@163.com (Y.R.); gaoli03@caas.cn (G.L.); Jason.White@ct.gov (J.C.W)

Summary

13 pages, 12 figures, 5 paragraph, 8 table.

Content

Section 1. Supplementary Methods S1

Chemicals and nanomaterial characterization. S1

Soil characterization measurement. S1

Section 2. Supplementary Figure S2

Supplementary Fig. 1 .... S2

Supplementary Fig. 2 .... S2

Supplementary Fig. 3 .... S3

Supplementary Fig. 4 .... S3

Supplementary Fig. 5 .... S4

Supplementary Fig. 6 .... S5

Supplementary Fig. 7 .... S5

Supplementary Fig. 8 .... S6

Supplementary Fig. 9 .... S7

Supplementary Fig. 10 .... S7

Supplementary Fig. 11 .... S8

Supplementary Fig. 12 .... S8

Section 3. Supplementary Table S9

Supplementary Table 1 .... S9

Supplementary Table 2 .... S9

Supplementary Table 3 .... S9

Supplementary Table 4 .... S9

Supplementary Table 5 .... S9

Supplementary Table 6 .... S11

Supplementary Table 7 .... S12

Supplementary Table 8 .... S12

Reference S13

Section 1. Supplementary Methods

Chemicals and nanomaterial characterization.

The MoS_2 NPs (99.99%, 80-100 nm) was obtained from Guangdong Nanuo Materials Technology Co., Ltd (Guangdong, China). MoS_2 Bulk, Na_2MoO_4 and all the other chemicals are purchased from Millipore Sigma. MoS_2 NS synthesized using previously reported methods Wang, et al. [1,2]. The MoS_2 (Millipore Sigma, China) was added to a hexane solution of 1.6 M n-butyllithium and stirred for 48 h under a stream of nitrogen. The product was then washed 3 times with hexane to remove lithium reagents and by-products. The product was sonicated for 1 h and then the unexfoliated MoS_2 material was removed by centrifugation at 500 rpm for 10 min to obtain a well-dispersed MoS_2 nanosheet dispersion. LiOH was removed from the dispersion by dialysis. The morphology and primary size of MoS_2 NPs, MoS_2 NS and MoS_2 Bulk were characterized by scanning electron microscopy (SU8020, HITACHI, Japan). The suspension of MoS_2 material (50 mg/L) was prepared in deionized water, subjected to 10 minutes of sonication (50 Hz, 220W), and then analyzed with a Zetasizer (Nano ZS90, Malvern, UK). The thickness of MoS_2 NS was analyzed by atomic force microscopy (Dimension Icon, Bruker AXS, Germany).

Soil characterization measurement.

Soil mechanical composition was determined by hydrometer method. Soil pH is glass electrode method (soil-water ratio is 2. 5:1). SOM was measured by Walkley-Black et al[3]; The content of total nitrogen in soil was determined by Kay et al[4]. Freeze-dried soil was ground into fine powders and digested in a mixture of $HF-HCl-HNO_3$(v/v/v: 1:1:3) in a microwave digestion system (MARS 6, UK). Elements (P, K, S, and Mo) were then determined by ICP-MS (Thermo Scientific). Soil (GBW 07406) were used as standard reference materials as described by Ping, et al. [5]. Calibration standards of known concentrations (0.01-100 ppm) were used for quantification.

Section 2. Supplementary Figure

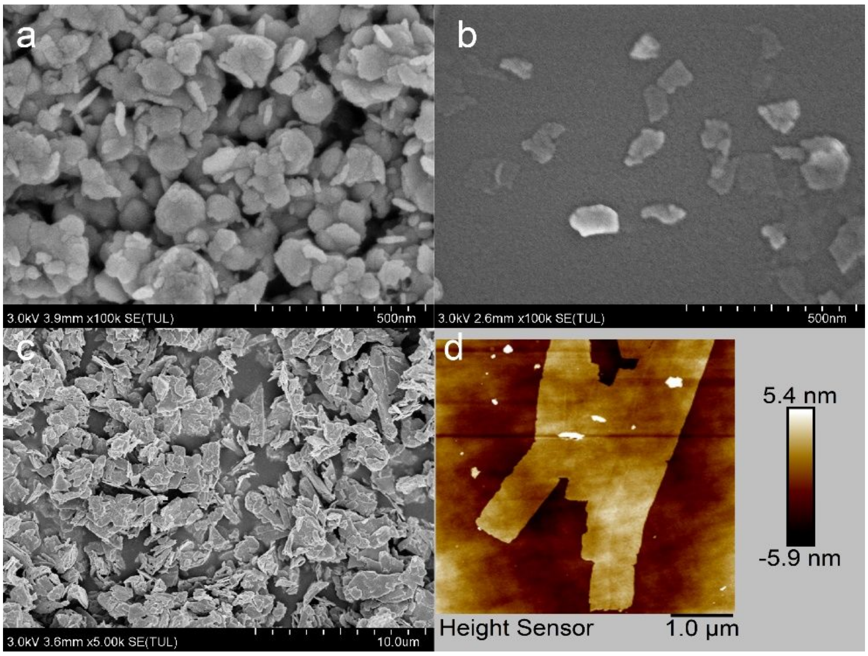

Supplementary Fig. 1 Morphology characterization of MoS_2. **(a-c)** SEM images of MoS_2 NPs (a), MoS_2 NS (b) and MoS_2 Bulk (c). **(d)** The thickness analysis of MoS_2 NS by AFM.

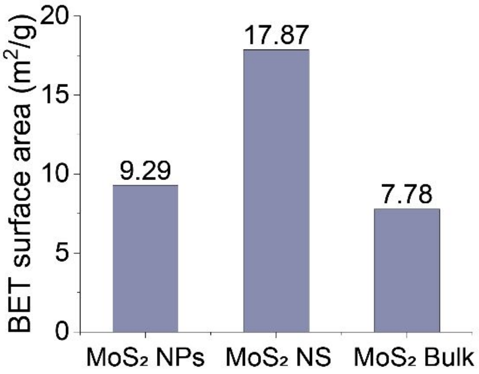

Supplementary Fig. 2 BET surface area of MoS_2 NPs, MoS_2 NS and MoS_2 Bulk.

MoS_2 NPs and MoS_2 NS have similar average lateral size (106.8 nm and 115.6 nm), the main difference is in the thickness: MoS_2 NPs is 20.1 nm thick, while MoS_2 NS is only 4.3 nm thick. MoS_2 NPs are more close to 3D particles while MoS_2 NS are more close to 2D sheet. The diameter of the MoS_2 Bulk average lateral size is 2.6 μm and the thickness is 121.1 nm (**Supplementary Fig. 1**). In our previous work, we characterized materials using XPS and BET surface area[6]. The results showed that the ratio of 1T/2H for MoS_2 NPs, MoS_2 NS and MoS_2 Bulk were 0.71,1 and 0.29, and the lattice oxygen were 10.9%, 34.8% and 6.1%, respectively (**Supplementary Table 6**). The specific surface areas

of MoS_2 NPs, MoS_2 NS and MoS_2 Bulk were 9.29,17.87 and 7.87 m^2/g, respectively (**Supplementary Fig. 2**).

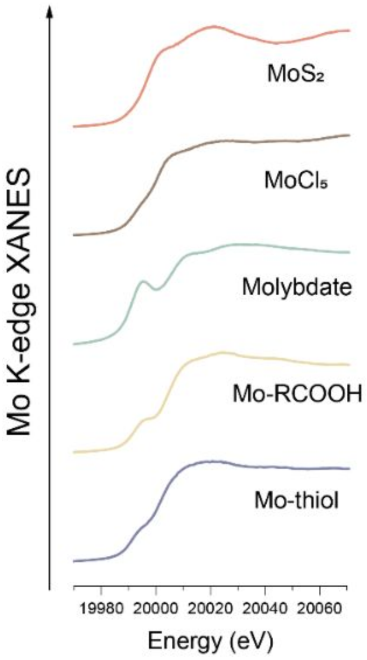

Supplementary Fig. 3 Mo K-edge XANES of reference materials including MoS_2, $MoCl_5$, Na_2MoO_4, Mo-RCOOH and Mo-thiol.

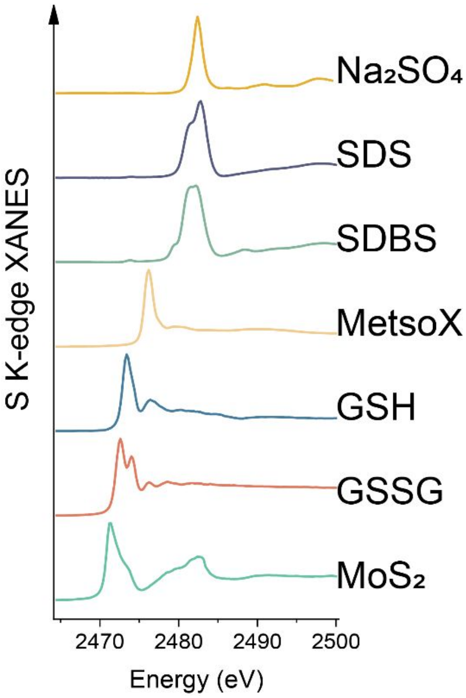

Supplementary Fig. 4 Sulfur K-edge XANES of reference materials including MoS_2, oxidized glutathione (GSSG), glutathione (GSH), Methionine sulfoxide (MetsoX), sodium dodecyl benzene sulfonate (SDBS), Sodium dodecyl sulfate (SDS)and Na_2SO_4.

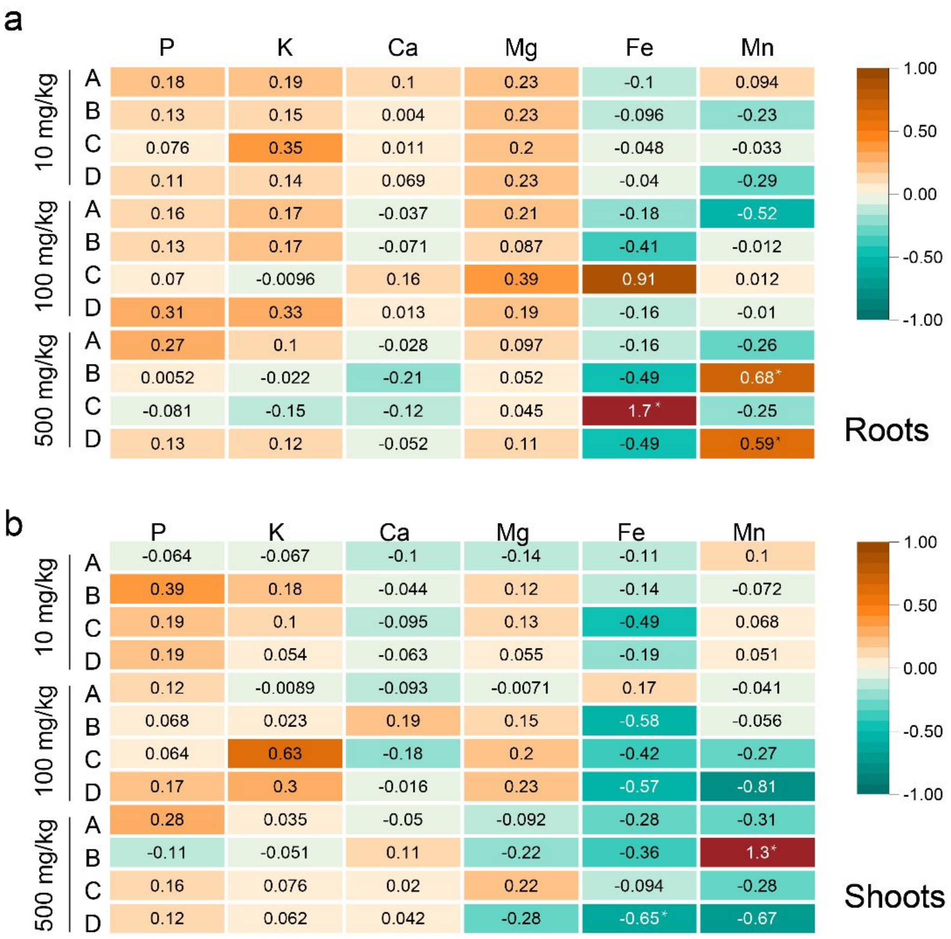

Supplementary Fig. 5 (a, b) Heatmap showing the homeostasis of inorganic nutrients in roots and shoots at 30 days. Numbers indicate the fold change of elemental content compared with the control group. A, B, C and D represent treatment groups MoS_2 NPs, MoS_2 NS, MoS_2 Bulk and Na_2MoO_4, respectively. Statistical significance was tested with one way ANOVA analysis with a Tukey's test. Numbers indicate the fold change of elemental content compared with the control group (n=6). * represent significant difference compared with control at $P < 0.05$.

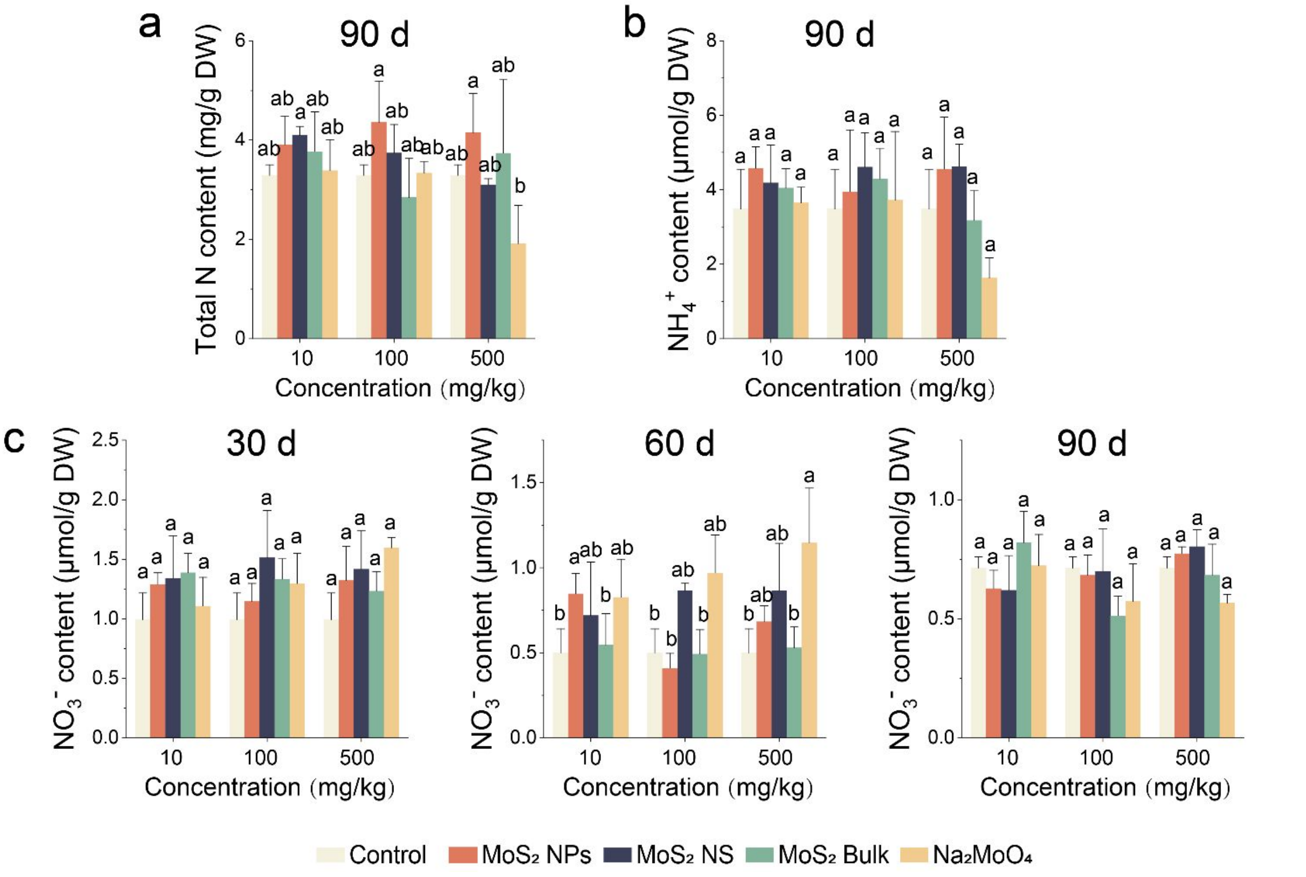

Supplementary Fig. 6 (a, b) Total nitrogen content and NH_4^+ content in nodules at 90 days. (c) NO_3^- content in nodules at 30, 60 and 90 days. Different lowercase letters indicate significant difference between groups.

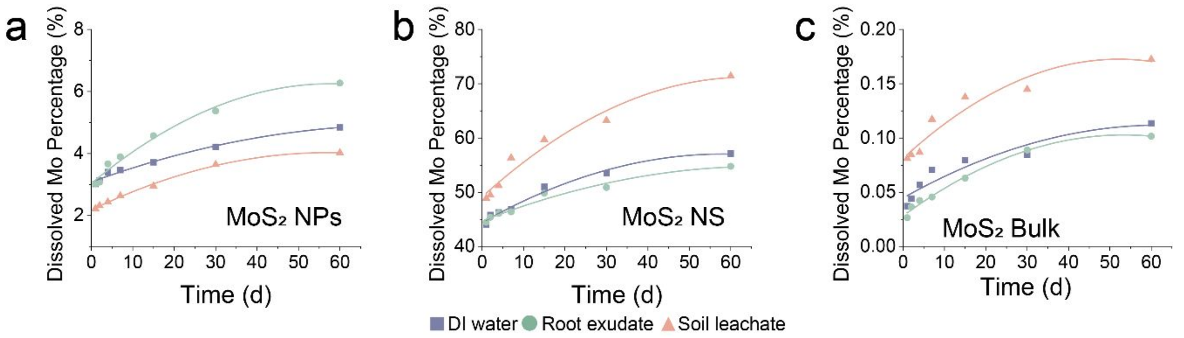

Supplementary Fig. 7 Dissolved Mo percentage from MoS_2 NPs (a), MoS_2 NS (b) and MoS_2 Bulk (c) in DI water, root exudates and soil leachate over 60 days.

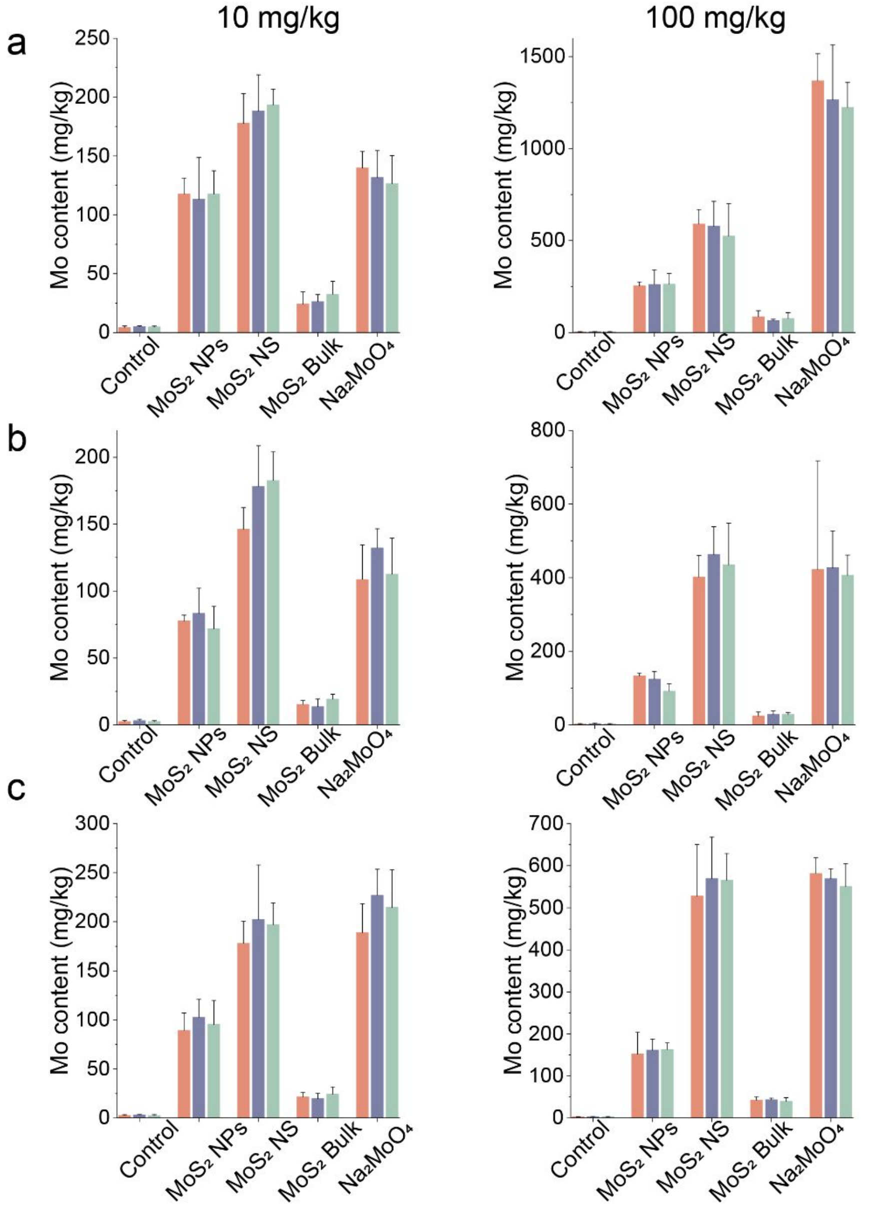

Supplementary Fig. 8 Mo content of soybean roots (a), shoots (b) and nodules (c) treated with 10 and 100 mg/kg four material and control group at 30 d, 60 d and 90 d.

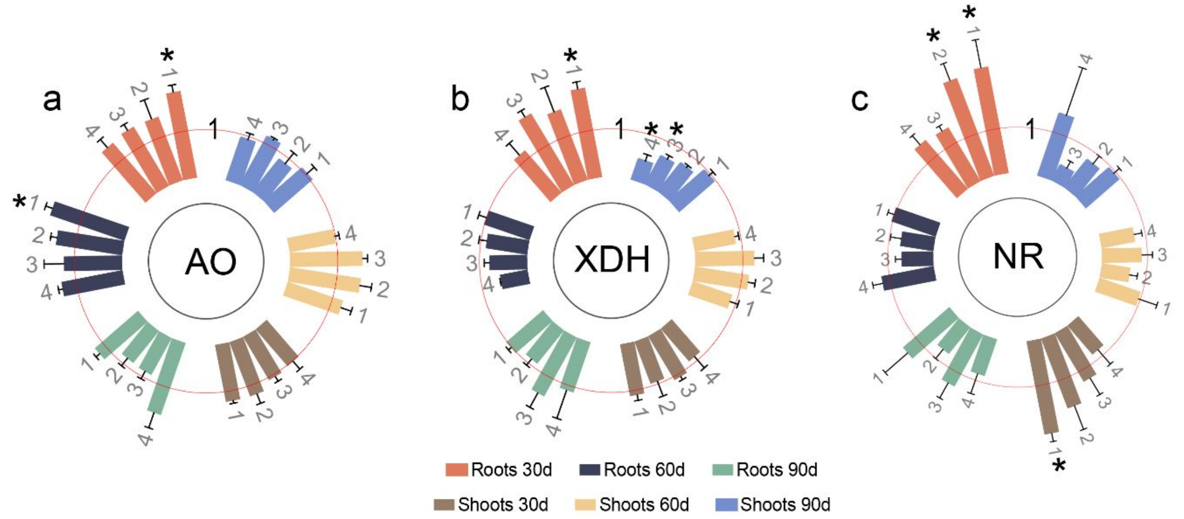

Supplementary Fig. 9 (a-c) Effects of four materials at 500 mg/kg on molybdenum enzymes, including AO, XDH and NR. The bar is a fold change relative to the control group. 1, 2, 3 and 4 represent treatments of 500 mg/kg MoS2 NPs, MoS2 NS, MoS2 Bulk and Na2MoO4, respectively. The data are shown as the mean ± SD (n=6). Statistical significance was tested with one-way ANOVA analysis with a Tukey's test. * represents P < 0.05.

Mo affects plant physiological and biochemical processes through Mo enzymes. Mo enzyme activities were measured in soybean at 30 days (nutritional growth stage), 60 days (R3, pod initiation) and 90 days (R6, bulge stage) (**Supplementary Fig. 9**). The results showed that MoS_2 NPs treatment increased AO and XDH activities in roots by 84% and 90% at 30 days, NR activities in roots and shoots by 138% and 108% at 30 days and AO activities by 64% at 60 days, respectively. MoS_2 NS treatment increased NR activity in soybean roots by 129% at 30 days. Both MoS_2 Bulk and Na_2MoO_4 reduced shoots XDH activity at 90 days.

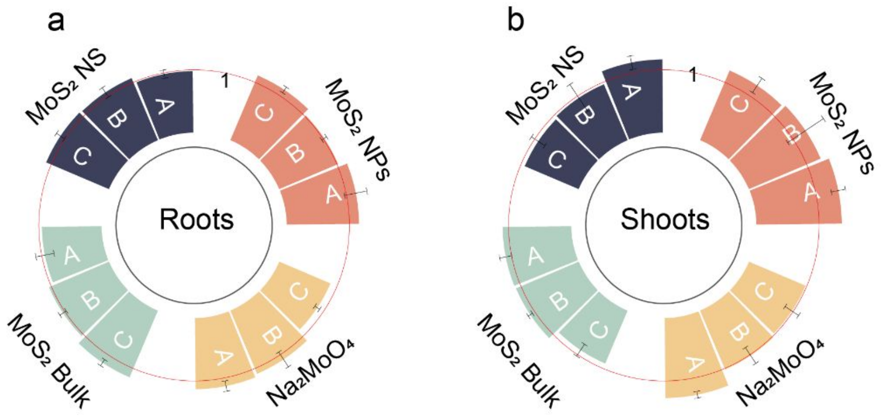

Supplementary Fig. 10 N content of soybean roots (a) and shoots (b) treated with four material and control group at 30 d, 60 d and 90 d. A, B, and C represent 10, 100 and 500 mg/kg, respectively.

Compared with the control group, the N content of soybean roots treated with 10 mg/kg, 100 mg/kg and 500 mg/kg MoS_2 NPs increased by 0.15, 0.02 and 0.12 times, respectively, and that of shoots increased by 0.36, 0.24 and 0.20 times, respectively (**Supplementary Fig. 10**). Nitrogen drives the

growth and development of plant trophic stages, and the increase in grain yield and protein content is quantitatively dependent on N assimilation in soybean. The increase in grain yield and protein content was quantitatively dependent on N assimilation in soybean.

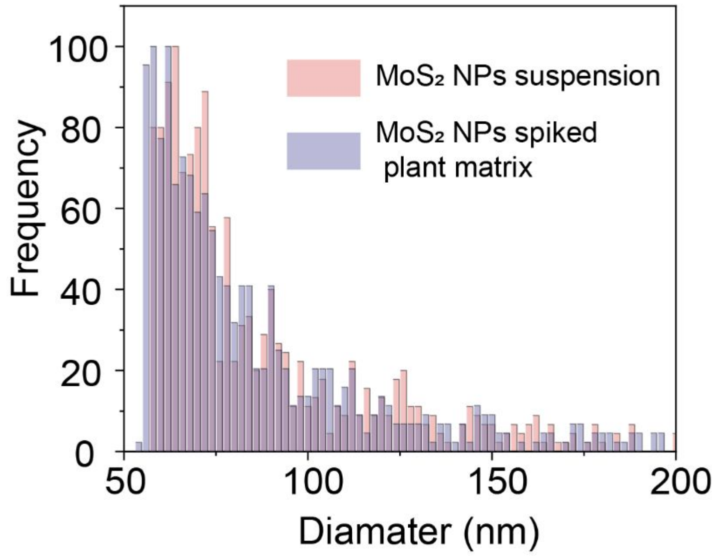

Supplementary Fig. 11 The particle size distribution histograms of MoS_2 NPs in suspension and spiked plant matrix suspension in a preliminary test.

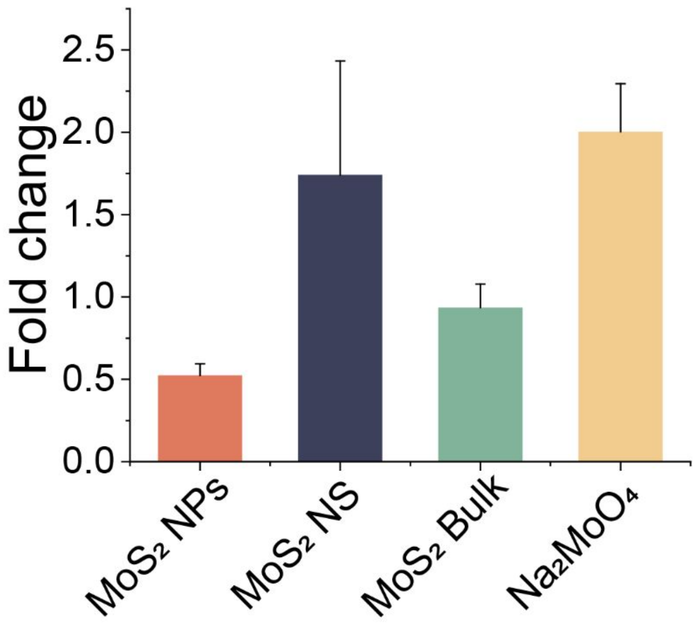

Supplementary Fig. 12 MDA content in nodule after treatment with 500 mg/kg of the Mo based materials for 60 d. The height of the bar indicates the fold change relative to the control.

Section 3. Supplementary Table

Supplementary Table 1. Soil parameters used in the experiment.

soil mechanical composition	pH	Electrical conductivity (μS/cm)	organic matter (g/kg)	Total N (g/kg)	Total P (mg/kg)	Total K (mg/kg)	Total S (mg/kg)	Total Mo (mg/kg)
silt 14%, sand 84%, clay 4%	6.76	801	3.87 %	1.57	49.6	131.2	350.23	1.20

Supplementary Table 2. Hydrodynamic size and Zeta potential of the MoS_2 NPs, MoS_2 NS and MoS_2 Bulk.

	MoS_2 NPs	MoS_2 NS	MoS_2 Bulk
Zeta potential [7]	-40.8	-34.6	-20.8
Hydrodynamic diameter (nm)	275.0 ±8.46	141.4±3.6	3233.3±282.6

Supplementary Table 3. Limit of detection, precision and recovery data of ICP-MS for the selected elements.

Elements	Limits of Detection (μg/L)	Recovery	Correlation coefficient (R^2)	RSD
Mo	0.017	98.1%	0.9999	1.7
K	0.043	102.8%	0.9998	2.5
Ca	0.048	98.5%	0.9998	2.5
Mg	0.033	101.8%	0.9999	2.1
P	0.036	98.0%	0.9999	1.9
Fe	0.042	101.3%	0.9997	2.6
Mn	0.029	101.7%	0.9996	2.3
Cu	0.064	97.3%	0.9997	1.8
Zn	0.048	97.1%	0.9998	2.6

Supplementary Table 4. Instrument parameters for SP-ICP-MS measurements.

Instrumental parameters	parameter values
ICPMS model	Aglient 7900
Nebulizer	MicoMist (borosilicate glass)
Spray chamber	Scott-type double-pass (quartz)
RF power (W)	1550
Nebulizer gas flow (L/min)	1.05
Sample flow rate (mL/min)	0.401

Supplementary Table 5. Fitting parameters from LCF analysis of XANES spectra of Mo and S of sample.

	Treatments	Sample	Time (day)	R-factors	Chi-square
Mo	MoS_2 NPs	Pot soil	30	0.0004532	0.00319
			60	0.0035996	0.01984
		Rhizosphere soil	30	0.0024217	0.01287

			60	0.0021749	0.01695
		Soybean Root	30	0.0019941	0.0337
			60	0.0016638	0.0269
			90	0.0001927	0.00165
		Soybean nodule	30	0.0017126	0.03076
			60	0.0013169	0.02428
			90	0.0003314	0.00292
		Soybean shoot	30	0.0046919	0.10011
			60	0.0026413	0.05544
			90	0.0003591	0.00313
	MoS_2 NS	Pot soil	30	0.0007878	0.00511
			60	0.0013026	0.00785
		Rhizosphere soil	30	0.0006481	0.004
			60	0.0007825	0.00496
		Soybean Root	30	0.0015385	0.02708
			60	0.0041787	0.06265
			90	0.0003301	0.00322
		Soybean nodule	30	0.0015681	0.02501
			60	0.001476	0.02443
			90	0.00297	0.00521
		Soybean shoot	30	0.0014491	0.0257
			60	0.0101841	0.13831
			90	0.0003261	0.00297
S	MoS_2 NPs	Soybean Root	30	0.010796	0.53342
			60	0.0098488	0.48435
			90	0.0024102	0.2245
		Soybean nodule	30	0.0039391	0.18023
			60	0.0099337	0.41483
			90	0.0088944	0.41688
		Soybean shoot	30	0.0048254	0.21699
			60	0.008079	0.35779
			90	0.0080951	0.37494
	MoS_2 NS	Soybean Root	30	0.0049418	0.61516
			60	0.0021446	0.0981
			90	0.0019947	0.36334
		Soybean nodule	30	0.0041747	0.23253
			60	0.0090365	0.45451

			90	0.0073467	0.41142
		Soybean shoot	30	0.0046807	0.21569
			60	0.0098124	0.40638
			90	0.0060792	0.2619
	MoS_2 Bulk	Soybean Root	30	0.0047628	0.21141
			60	0.0031525	0.17863
			90	0.0039487	0.18269
		Soybean nodule	30	0.0051654	0.23425
			60	0.0067093	0.30371
			90	0.0068268	0.29856
		Soybean shoot	30	0.0063735	0.3005
			60	0.0047553	0.23226
			90	0.0096625	0.36968
	Control	Soybean Root	30	0.011116	0.66117
			60	0.0027689	0.08654
			90	0.0018501	0.10709
		Soybean nodule	30	0.004329	0.19261
			60	0.0089614	0.37501
			90	0.0050186	0.2349
		Soybean shoot	30	0.00744	0.29358
			60	0.0061509	0.25118
			90	0.0039432	0.20238

Supplementary Table 6. The yield of soybean grain.

Treatments		Yield (g grains/plant)
MoS_2 NPs	10	3.34
	100	4.51
	500	3.64
MoS_2 NS	10	3.75
	100	3.49
	500	3.59
MoS_2 Bulk	10	2.36
	100	2.99
	500	3.58
Na_2MoO_4	10	2.77
	100	3.46

	500	2.72

Supplementary Table 7. XPS fitting results

	1T / 2H ratio	Lattice oxygen ratio
MoS_2 NPs	0.71	10.9%
MoS_2 NS	1	34.8%
MoS_2 Bulk	0.29	6.1%

Supplementary Table 8. The average particle size of the MoS_2 NPs in soybean tissues

	Treatment	10 mg/kg			100 mg/kg			500 mg/kg		
	Time (day)	30	60	90	30	60	90	30	60	90
Particle size (nm)	Roots	32.7	36.7	37.1	39.9	34.6	33.3	37.9	52.6	52.2
	Shoots	43.2	42.5	44.1	50.3	52.5	53.8	40.4	42.4	49.3
	Nodules	36.5	38.0	32.2	41.8	41.6	50.2	47	42.5	44

Reference

1. Wang, Z. Y.; von dem Bussche, A.; Qiu, Y.; Valentin, T. M.; Gion, K.; Kane, A. B.; Hurt, R. H., Chemical dissolution pathways of MoS2 nanosheets in biological and environmental media. *Environmental Science & Technology* **2016,** *50*, (13), 7208-7217.
2. Eda, G.; Yamaguchi, H.; Voiry, D.; Fujita, T.; Chen, M.; Chhowalla, M., Photoluminescence from chemically exfoliated MoS2. *Nano Letters* **2011,** *11*, (12), 5111-5116.
3. Roper, W. R.; Robarge, W. P.; Osmond, D. L.; Heitman, J. L. J. S. e. S. o. A. J., Comparing Four Methods of Measuring Soil Organic Matter in North Carolina Soils. Soil Science Society of America Journal **2019,** 83, 466-474.
4. Bremner, J. M., Total nitrogen. **1965**.
5. Ping, Z.; Huiling, Z.; Wensheng, S. J. J. o. E. S., Biotransfer of heavy metals along a soil-plant-insect-chicken food chain: Field study. *Journal of Environmental Sciences* **2009,** *21*, (006), 849-853.
6. Li, M.; Zhang, P.; Guo, Z.; Cao, W.; Gao, L.; Li, Y.; Tian, C. F.; Chen, Q.; Shen, Y.; Ren, F.; Rui, Y.; White, J. C.; Lynch, I., Molybdenum Nanofertilizer Boosts Biological Nitrogen Fixation and Yield of Soybean through Delaying Nodule Senescence and Nutrition Enhancement. *ACS nano* **2023**, 17, (15), 14761-14774.
7. Wyrzykowska, E.; Mikolajczyk, A.; Lynch, I.; Jeliazkova, N.; Kochev, N.; Sarimveis, H.; Doganis, P.; Karatzas, P.; Afantitis, A.; Melagraki, G.; Serra, A.; Greco, D.; Subbotina, J.; Lobaskin, V.; Bañares, M. A.; Valsami-Jones, E.; Jagiello, K.; Puzyn, T., Representing and describing nanomaterials in predictive nanoinformatics. *Nature Nanotechnology* **2022,** *17*, (9), 924-932.
